# Norditerpenoids with Selective Anti-Cholinesterase Activity from the Roots of *Perovskia atriplicifolia* Benth.

**DOI:** 10.3390/ijms21124475

**Published:** 2020-06-23

**Authors:** Sylwester Ślusarczyk, F. Sezer Senol Deniz, Renata Abel, Łukasz Pecio, Horacio Pérez-Sánchez, José P. Cerón-Carrasco, Helena den-Haan, Priyanka Banerjee, Robert Preissner, Edward Krzyżak, Wiesław Oleszek, Ilkay E. Orhan, Adam Matkowski

**Affiliations:** 1 Department of Pharmaceutical Biology and Botany, Wroclaw Medical University, 50556 Wroclaw, Poland; sylwester.slusarczyk@umed.wroc.pl (S.Ś.); renata.abel@umed.wroc.pl (R.A.); 2 Department of Pharmacognosy, Faculty of Pharmacy, Gazi University, 06330 Ankara, Turkey; fssenol@gazi.edu.tr (F.S.S.D.); iorhan@gazi.edu.tr (I.E.O.); 3 Structural Bioinformatics Group, Institute for Physiology & ECRC, Charité University Medicine, 10115 Berlin, Germany; priyanka.banerjee@charite.de (P.B.); robert.preissner@charite.de (R.P.); 4 Department of Biochemistry and Crop Quality, IUNG-Institute of Soil Science and Plant Cultivation, 24100 Puławy, Poland; lpecio@iung.pulawy.pl (Ł.P.); wo@iung.pulawy.pl (W.O.); 5 Structural Bioinformatics and High Performance Computing Research Group, Universidad Católica San Antonio de Murcia (UCAM), 30107 Murcia, Spain; hperez@ucam.edu (H.P.-S.); helenadenhaan@gmail.com (H.d.-H); 6 Reconocimiento y Encapsulación Molecular (REM), Universidad Católica San Antonio de Murcia (UCAM), 30107 Murcia, Spain; jpceron@ucam.edu; 7 Department of Inorganic Chemistry, Wroclaw Medical University, 50556 Wroclaw, Poland; edward.krzyzak@umed.wroc.pl

**Keywords:** tanshinones, cholinesterases, molecular docking, structure elucidation

## Abstract

Inhibition of cholinesterases remains one of a few available treatment strategies for neurodegenerative dementias such as Alzheimer’s disease and related conditions. The current study was inspired by previous data on anticholinesterase properties of diterpenoids from *Perovskia atriplicifolia* and other Lamiaceae species. The acetylcholinesterase (AChE) and butyrylcholinesterase (BChE) inhibition by the three new natural compounds—(1*R*,15*R*)-1-acetoxycryptotanshinone (**1**), (1*R*)-1-acetoxytanshinone IIA (**2**), and (15*R*)-1-oxoaegyptinone A (**3**)—as well as, new for this genus, isograndifoliol (**4**) were assessed. Three of these compounds exhibited profound inhibition of butyrylcholinesterase (BChE) and much weaker inhibition of acetylcholinesterase (AChE). All compounds (**1**–**4**) selectively inhibited BChE (IC_50_ = 2.4, 7.9, 50.8, and 0.9 µM, respectively), whereas only compounds **3** and **4** moderately inhibited AChE (IC_50_ 329.8 µM and 342.9 µM). Molecular docking and in silico toxicology prediction studies were also performed on the active compounds. Natural oxygenated norditerpenoids from the traditional Central Asian medicinal plant *P. atriplicifolia* are selective BChE inhibitors. Their high potential makes them useful candidate molecules for further investigation as lead compounds in the development of a natural drug against dementia caused by neurodegenerative diseases.

## 1. Introduction

Acetylcholinesterase (AChE) inhibitors such as donepezil, galanthamine, rivastigmine, and tacrine are the most prescribed medications used to slow down the progression of cognitive decline in neurodegenerative diseases [1]. Butyrylcholinesterase (BChE)—another member of the cholinesterase family—is also postulated to upsurge in the late stages of Alzheimer’s disease and to accumulate in the amyloid plaques (Aβ) [2]. As such, it is also regarded as a feasible target for anti-dementia drug discovery. There are reports of BChE inhibitors acting in a similar way to anti-AChE remedies in slowing down the development of dementia symptoms [3]. Some of the clinically used drugs such as rivastigmine inhibit both forms, which are products of different genes but have similar molecular forms and active sites. At present, there is no established approach to prevent or cure Alzheimer’s disease, therefore, symptomatic treatment of cognitive deterioration, such as disturbances in memory and perception, using ChE inhibitors is still an important therapeutic opportunity [4,5]. Medicinal plants and natural compounds remain a rich resource of such inhibitors among which diterpenoids deserve considerable attention. Cognitive improvement via cholinesterase inhibition can also have broader applications as a form of complementary therapy, for example, during distress or as an aid in smoking cessation [6,7].

Another important, non-medical application of anticholinesterase substances from plants is their potential as natural insecticides that can provide an alternative to environmentally harmful chemicals such as the recently banned neonicotinoids and many other chemical classes. The mechanism is based on the disruption of cholinergic signaling in the nervous system of an insect, leading to paralysis. It is a target for such chemical insecticides as carbamates and organophosphorus compounds [8]. Several species from the Lamiaceae have been studied from this viewpoint [9,10].

The class of diterpenoids known as tanshinones contains norabietanoid quinones that are red or orange in color. *Salviae miltiorrhizae radix* (danshen), an important traditional Chinese herb, is the richest and most studied source of them [11]. Tanshinones exhibit numerous pharmacological activities such as anticancer, anti-aging, anti-convulsant, and antioxidative by activating nuclear erythroid 2-related factor 2 (Nrf2)**,** etc. [11,12,13,14]. Tanshinone IIA is undergoing a phase III clinical trial for cardiovascular conditions [15,16]. Besides *S. miltiorrhiza*, several other species of the genus *Salvia* L. along with a few other Lamiaceae genera contain tanshinones in various proportions. One of these, *Perovskia atriplicifolia* Benth. (Lamiaceae), a Central Asian species, is cultivated worldwide as an ornamental called “Russian sage”. However, a recent botanical classification included *Perovskia* and all other tanshinone-containing genera in the extended *Salvia* genus, renaming *P. atriplicifolia* as *Salvia yangii* B.T.Drew [17]. Probably, this very close taxonomic relationship is reflected in the phytochemical similarities between *S. miltiorrhiza* and other sages and the plant studied here. As we previously reported, *P. atriplicifolia* contains a high amount of norditerpenoids, with cryptotanshinone being the major constituent [18]. However, this major compound possesses only moderate cholinesterase inhibitory activity [19,20]. Although a large number of ChE inhibitors have been reported from plants, only some of the known tanshinones and related compounds have been investigated for their potential to inhibit ChEs [19,21]. This was the motivation for a search into the natural diversity of diterpenoids in *Perovskia* roots and for screening the newly obtained compounds for their potential as anticholinesterase drugs.

In the present work, an isolation protocol was developed for three new diterpenoids and one rare compound known from other species. Next, their in vitro AChE and BChE inhibitory activities were assessed and complemented by molecular docking studies to understand the mechanisms of interaction between the isolated compounds and the enzymes.

## 2. Results and Discussion

**Figure 1:**
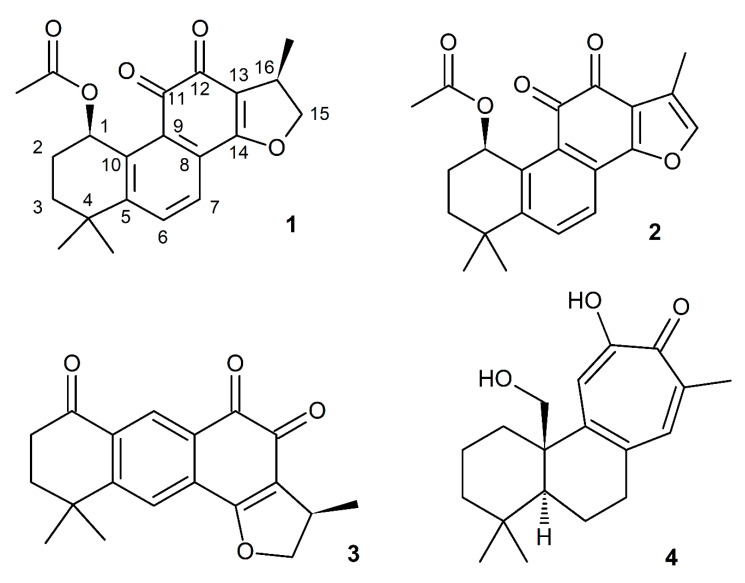
Structures of compounds **1**–**4** isolated from roots of *Perovskia atriplicifolia.*

**Figure 2:**
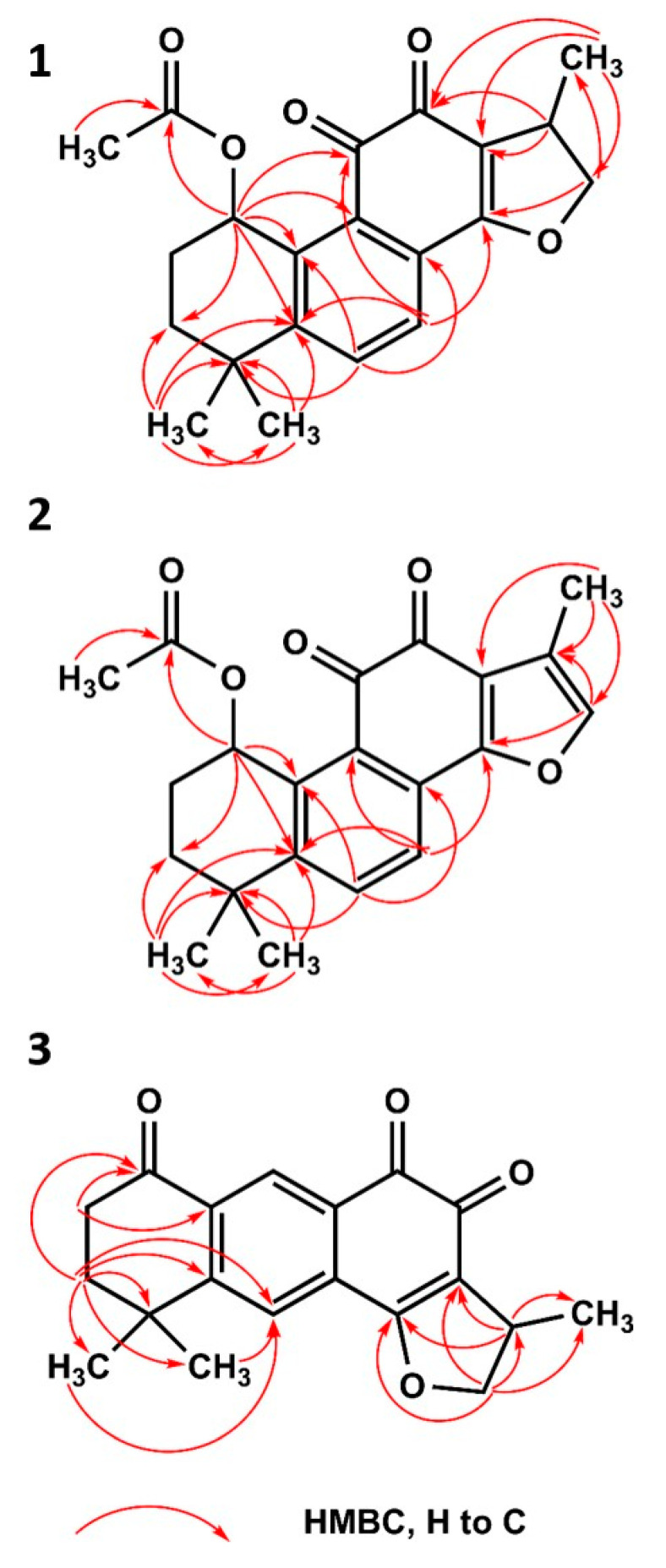
HMBC correlations of compounds **1**–**3**.

The ongoing investigation of *P. atriplicifolia* phytochemistry has led to the isolation and elucidation of the structure of three new natural compounds and one compound new to this species. The *n*-hexane extract was fractionated using CC followed by semipreparative RP-HPLC to afford (1*R*,15*R*)-1-acetoxycryptotanshinone (**1**), (1*R*)-1-acetoxytanshinone IIA (**2**), (15*R*)-1-oxoaegyptinone A (**3**), and, new for this species, isograndifoliol (**4**). The latter was previously obtained among 14 new norditerpenoids from a related *Salvia* species [22,23]. This compound, although included in the phytochemistry literature as a sensu lato abietane terpenoid, has a rearranged tricyclic ring structure, with a 7-carbon ring replacing a 6-carbon and a furan ring in typical tanshinones (Figure 1).

(1*R*,15*R*)-1-Acetoxycryptotanshinone (**1**) was obtained as a red amorphous powder. The molecular formula was established as C_21_H_22_O_5_ according to the high-resolution quadrupole time-of-flight mass spectrometer (HRQTOF-MS) ion (*m*/*z* 355.1538 [M+H]+, calculated for C_21_H_23_O_5_: 355.1540) and **^13^**C NMR data. The UV spectrum showed absorption maxima at 225, 270, and 441 nm. **^1^**H and **^13^**C NMR spectra showed 21 signals that were identified as four methyls resonating at δ_H_ 1.99, 1.39, 1.28 (each 3H, *s*), and a doublet at δ 1.32 (*J* = 6.8 Hz, 3H), two CH_2_ carbons at δ_H_ 1.93 (1H, *td*, *J* = 13.3, 2.6 Hz)/1.59 (1H, *ddd*, *J* = 13.3, 5.5, 2.5 Hz), 2.13–2.19 (1H, *m*)/2.02 (1H, *tdd*, *J* = 14.0, 3.5, 2.5 Hz; δ_C_ 33.1, 25.5), and an oxygenated methylene at δ_H_ 4.96 (1H, *t*, *J* = 9.4 Hz)/4.42 (1H, *dd*, *J* = 9.3, 6.2 Hz; δ_C_ 83.2), four CH carbons [two aromatic at δ_H_ 7.84 (1H, *d*, *J* = 7.7 Hz), 7.67 (1H, *d*, *J* = 7.8 Hz; δ_C_ 134.9, 126.4), at δ_H_/δ_C_ 3.48–3.58 (1H, *m*)/35.7, and an oxygenated methine at δ_H_/δ_C_ 6.40 (1H, *t*, *J* = 3.4 Hz)/68.7], three carbonyls (δ_C_ 184.5, 176.3—forming an *o*-quinone moiety—and 171.8), one enol carbon (δ_C_ 172.7), and six quaternary carbon atoms subdivided in a tetrasubstituted benzene ring (δ_C_ 154.2, 138.1, 130.1, 128.3), one olefinic (δ_C_ 119.6), and one aliphatic (δ_C_ 35.9) carbon atom (Table 1). Comparison of the NMR data of 1 with those of the known 1β-hydroxycryptotanshinone indicated that they possessed similar basic structures, except for the presence of an acetyl group in 1. (Figure S2, Supplementary Materials and Sairafianpour et al. [24]) The presence of the acetyl group was confirmed by the HMBC correlations (Figure 2) from the methyl group (δ_H_ 1.99) and H-1 (δ_H_ 6.40) to the carbonyl carbon (δ_C_ 171.8), and it was additionally supported by a significant (Δδ +4.8) deshielding of C-1 (δ_C_ 68.7) and significant (Δδ −2.7, −1.5, and −4.9) shielding of C-2/C-11/C-10 (δ_C_ 25.5, 184.5, and 138.1), respectively, compared to 1β-hydroxycryptotanshinone. The presence of an *ortho*-quinone moiety was apparent from the long-range (**^4^**J) correlations from H-1/H-7 (δ 6.40/7.67) to C-11 (δ 184.5), from H-17 (δ 1.32) to C-12 (δ 176.3), and **^3^**J correlation from H-15 (δ 3.48–3.58) to C-12. On the basis of these data, a 2D structure of (1) was established. It can be assumed that 1 is a biosynthetic derivative of 1β-hydroxycryptotanshinone because it has the same absolute configuration at C-1 and C-15, i.e., 1*R*,15*R,* which was confirmed by the comparison of experimental and calculated electronic circular dichroism (ECD) curves (Figure S1A), and specific rotations of similar signs; 1 ([α]_D_ = +133.0, CHCl_3_), and 1β-hydroxycryptotanshinone ([α]_D_ = +235, CHCl_3_) [23]. Thus, the structure of compound **1** was established as (1*R*,15*R*)-1-acetoxycryptotanshinone.

(1*R*)-1-Acetoxytanshinone IIA (**2**) was obtained as red needle-shaped crystals. The molecular formula of 2 was established as C_21_H_20_O_5_ according to its HRQTOF-MS (*m*/*z* 353.1385 [M+H]^+^, calculated for C_21_H_21_O_5_: 353.1384) and **^13^**C NMR data. The UV spectrum of 2 revealed absorption maxima at 221, 250, 271 and 460 nm. The detailed analysis of **^1^**H and **^13^**C NMR spectra of 2, similarly to 1, showed 21 signals that were classified as four methyls resonating at δ_H_ 2.01, 1.41, 1.31 (each 3H, *s*), and a doublet at δ_H_ 2.23 (*J* = 1.2 Hz, 3H), two CH_2_ carbons at δ_H_ 1.93 (1H, *td*, *J* = 13.1, 2.4 Hz)/1.58 (1H, *ddd*, *J* = 12.9, 5.6, 2.8 Hz), 2.13–2.19 (1H, *m*)/2.01 (1H, *tt*, *J* = 14.1, 2.6 Hz; δ_C_ 33.2, 25.6), four CH carbons—three aromatic at δ_H_ 7.89 (1H, *d*, *J* = 8.3 Hz), 7.80 (1H, *d*, *J* = 8.3 Hz) and 7.48 (1H, *q*, *J* = 1.3 Hz; δ_C_ 135.7, 124.2, 143.6), and one oxygenated methine at δ_H_/δ_C_ 6.41 (1H, *t*, *J* = 3.4 Hz)/69.8—three carbonyls (δ_C_ 183.8, 176.3 [forming a 1,2-quinone moiety], and 172.2), one oxygenated aromatic carbon (δ_C_ 162.7), seven quaternary carbon atoms subdivided in a tetrasubstituted benzene ring (δ_C_ 151.9, 138.7, 129.6, 128.2), trisubstituted furan ring (δ_C_ 122.3, 121.1) and one aliphatic (δ_C_ 35.8) carbon atoms (Table 1). The NMR spectroscopic properties of 2 were similar to (1*S*)-hydroxytanshinone IIA (Figure S3, Supplementary Materials and Senol et al. [20]), indicating a similar chemical pattern, except for the presence of one acetyl group located at C-1 of 2. The site of acylation was confirmed by the HMBC correlations (Figure 2) from the methyl group (δ_H_ 2.01) and H-1 (δ_H_ 6.41) to carbonyl carbon (δ_C_ 172.2), and it was additionally supported by a significant (Δδ +4.9) deshielding of C-1 (δC 69.0) and significant (Δδ −2.6, −1.4 and −4.9) shielding of C-2/C-11/C-10 (δ_C_ 25.6, 183.8 and 138.7), respectively, compared to (1*S*)-hydroxytanshinone IIA. Based on these data, the 2D structure of 1-acetoxytanshinone IIA (**2**) was established. The absolute configuration of 2 was established by the comparison of experimental and calculated electronic circular dichroism (ECD) curves (Figure S1B). Unfortunately, the optical rotation of (1*S*)-hydroxytanshinone IIA was not reported, but the rotation of 2 ([α]_D_ = +203.0, CHCl_3_) may be compared with that of the related compound (1*R*)-hydroxymiltirone ([α]_D_ = +989.1, CHCl_3_) [25], thus, the structure of compound **2** was established as (1*R*)-1-acetoxytanshinone IIA.

(15*R*)-1-oxoaegyptinone A (**3**) was obtained as red needle-shaped crystals. The molecular formula of 3 was established as C_19_H_18_O_4_ according to its HRQTOF-MS data (*m*/*z* 311.1277 [M+H]^+^, calculated for C_19_H_19_O_4_: 311.1278) and **^13^**C NMR data. The UV spectrum of 3 showed absorption maxima at 224 and 290 nm. The **^1^**H and **^13^**C NMR spectra showed 19 signals that were classified as three methyls resonating at δ_H_ 1.352, 1.350 (each 3H, *s*), and a doublet at δ 1.347 (*J* = 6.9 Hz, 3H), two CH_2_ carbons at δ_H_ 2.10 (2H, *t*, *J* = 7.2 Hz) and 2.90 (2H, *t*, *J* = 7.2 Hz; δ_C_ 37.5, 37.3), and an oxygenated methylene at δ_H_ 5.01 (1H, *t*, *J* = 9.6 Hz)/4.48 (1H, *dd*, *J* = 9.5, 6.2 Hz; δ_C_ 83.6), three CH carbons [two aromatic at δ_H_ 7.78 (2H, overlapping); δ_C_ 131.7, 128.2, and one aliphatic at δ_H_/δ_C_ 3.54–3.64 (1H, *m*)/35.8], three carbonyls (δ_C_ 201.8, 184.9, and 178.9), one enol carbon (δ_C_ 172.0), and six quaternary carbon atoms subdivided in a tetra-substituted benzene ring (δ_C_ 157.9, 138.7, 134.2, 128.6), one olefinic (δ_C_ 120.4), and one aliphatic (δ_C_ 36.4) carbon atom (Table 1). The data of 3 suggested that it may be an isomer of 1-oxocryptotanshinone (Figure S4), with its core similar to aegyptinone A with one methyl group absent, and with an additional carbonyl carbon [26]. The presence of the latter was confirmed by the HMBC correlations (Figure 2) from H-2/H-3 (δ_H_ 2.90/2.10) to C-1 (δ_C_ 201.8), and chemical shifts of C-2/C-3 (δ_C_ 37.3/37.5) similar to those of 1-oxocryptotanshinone (36.4/37.6, respectively). However, a number of differences between 1-oxocryptotanshinone and 3 were observed. Namely, C-5, corresponding to C-10 in 1-oxocryptotanshinone (δ_C_ 138.7), experienced a significant high-frequency shift (Δδ +9.3), while C-9 was upfield shifted (Δδ -6.4). Additionally, significant (Δδ +9.1, both) deshielding of C-11/C-12 was observed (δ_C_ 184.9/178.9, respectively), compared to 1-oxocryptotanshinone. In the **^1^**H NMR spectrum, the *ortho-* coupled H-6/H-7 were replaced by a pair of *para-* protons at δ_H_ 7.78, and correlating in the HMBC spectrum with C-1/C-4/C-5/C-6/C-8/C-9/C-11/C-14 (δ_C_ 201.8./36.4/138.7/157.9/128.6/134.2/184.9/172.0). On the other hand, the HMBC spectrum of 1-oxocryptotanshinone showed no correlation to the C-11. The position of C-7 (δ_C_ 131.7) was evidenced from the long-range (**^4^**J) correlation (from H-3) visible in the HMBC spectrum tuned to ^n^J_CH_ = 3 Hz. Based on these data, a 2D structure of 3 was established. The absolute stereochemistry of C-15 was established as 15*R*, based on the comparison of experimental and calculated electronic circular dichroism (ECD) curves (Figure S1C), and the optical rotation ([α]_D_ = −3.8, CHCl_3_), similar to close analogs of 3, i.e., aegyptinone A ([α]_D_ = −102.0, CHCl_3_) and 1-oxocryptotanshinone ([α]_D_ = −59.3, CHCl_3_) [24,26,27]. This norabietane has a tricyclic structure rearranged in a linear form, thus resembling an anthraquinone. Therefore, some of its pharmacological properties could also be related to another phytochemical class of completely unrelated biosynthetic origin, namely anthraquinoids. This is the first record of a compound with a linear tricyclic skeleton in *P. atriplicifolia*, confirming this plant as a valuable source of unique diterpenoids, in addition to the previously isolated perovskatones and many other substituted variations of the abietane core [22,28]. By now, this type of structurally unique compound seems to be limited to some species of the extended *Salvia* genus and the knowledge on their pharmacological properties is scarce [29]. The quantities of these compounds estimated using HPLC in the roots are also sufficient (Table 2) to obtain suitable amounts for pharmacological testing. Isograndifoliol, which exceeded 0.3% of the dry root mass, is also likely to significantly contribute to the medicinal properties of the crude drug, in addition to the major tanshinones. 

**Figure 3:**
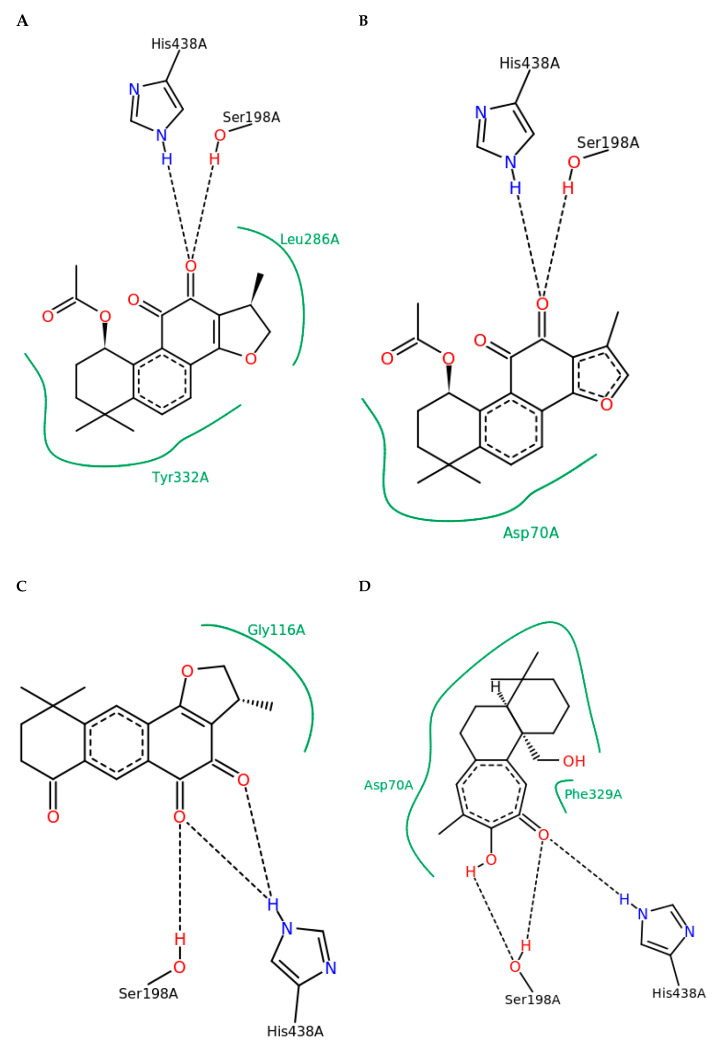
2D depiction of main interactions established between compounds **1**–**4** and BChE. The green continuous line represents hydrophobic interactions while the black dashed lines correspond to hydrogen bonds. (**A**) 2D depiction of main interactions established between compound **1** and BChE. (**B**) 2D depiction of main interactions established between compound **2** and BChE. (**C**) 2D depiction of main interactions established between compound **3** and BChE. (**D**) 2D depiction of main interactions established between compound **4** and BChE.

All four isolated norditerpenoids inhibited both forms of cholinesterase (Table 2). However, there were striking differences between both activity and specificity towards the forms. Compound **1** inhibited BChE activity by 95.89%, compound **2** by 85.34%, compound **3** by 87.34%, and isograndifoliol (**4**) by 98.6%, whilst inhibition of AChE was not more than 50%. Isograndifoliol (**4**) was the most potent and selective inhibitor of BChE, with IC_50_ below one micromole, whereas IC_50_ for AChE was above 340 µM (103.73 ± 4.49 µg·mL^−1^). Therefore, isograndifoliol ranks among the strongest non-nitrogenous BChE inhibitors from plant sources, compared to most values reported in the literature, which seldom drop below 10 µM [30]. Several norabietanoid compounds isolated from the very same species in our previous study were about three to four times weaker [20]. On the other hand, the two new tanshinone derivatives (1 and 2) were similarly as potent as the previously isolated ones, including both 1-hydroxy derivatives of major tanshinones (1*β*-OH-cryptotanshinone from *P. atriplicifolia* and (1*S*)-OH-tanshinone IIA from *Salvia glutinosa*), which are non-acetylated forms of **1** and **2**. It suggests a minor role of the acetyl moiety in this kind of activity. Molecular docking also supports this assumption, as no strong interactions of the acetyl group with the enzyme active site were observed for 1 and 2 (Figure 3A,B, activity against BChE). Indeed, a similar situation regarding the acetyl group was found for **3** and **4** (Figure 3C,D, activity against BChE). 

The new linear anthraquinone-like abietanoid (**3**) was less active but also less selective towards BChE, inhibiting AChE activity by 50% (IC_50_ = 102.30 ± 3.18 µg·mL^−1^; 329.8 µM), like isograndifoliol.

**Figure 4:**
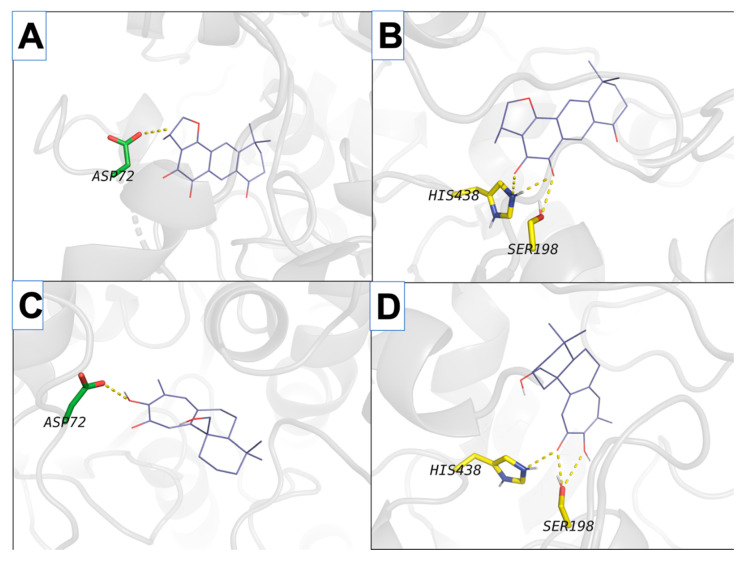
Interactions of isolated compounds (purple skeleton) that interact with both AChE and BChE, in transparent grey cartoon fashion. The left column (**A**,**C**) corresponds to AChE while the right column (**B**,**D**) corresponds to BChE. The first and second rows correspond to (*15R*)-1-oxoaegyptinone A (**A**,B) and isograndifoliol (**C**,**D**), respectively. Residues are colored according to their binding areas as yellow (catalytic triad) and green (peripheral anionic site). Hydrogen bonds established between compounds and depicted residues are shown with deep yellow dashed lines.

**Figure 5:**
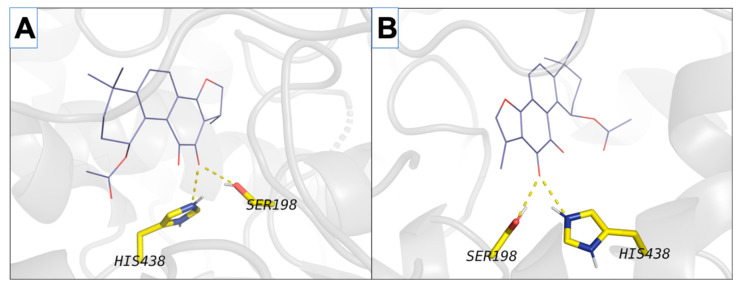
Interactions of isolated compounds from *Perovskia atriplicifolia* extracts (purple skeleton) that only interact with BChE, in transparent grey cartoon fashion. Shown compounds are (*1R,15R*)-1-acetoxycryptotanshinone (**A**), and (*1R*)-1-acetoxytanshinone IIA (**B**). Residues are colored according to their binding areas as yellow (catalytic triad) and green (peripheral anionic site). Hydrogen bonds established between compounds and depicted residues are shown with deep yellow dashed lines.

With the purpose of understanding the possible mechanisms of the marked in vitro ChE inhibitory activity at the molecular level, the molecular docking experiments were performed (Figure 4 and Figure 5).

All these compounds inhibit both targeted proteins with higher affinity to BChE than to AChE. Among them, isograndifoliol (compound **4**) exhibited the highest activity (IC_50_ 0.89 µM); it was also the most specific BChE inhibitor, despite an apparent overall structure similarity to galanthamine. One of the best-known active AChE inhibitors is tacrine, but it was withdrawn from the pharmaceutical market due to hepatotoxicity. Three other AChE inhibitors used in Alzheimer’s disease therapy are rivastigmine, donepezil and galantamine [31]. Interestingly, similar chemical structural features to tacrine (amino tetrahydroacridine) appear in compound **3**, with IC_50_ values 50.80 µM against BChE and 342 µM against AChE, which render this compound the least specific one among the four studied. The main difference is a furan ring in (15*R*)-1-oxoaegyptinone A (**3**) appearing instead of an amino moiety in the structure of tacrine. Aegyptinone A, an analog of (15*R*)-1-oxoaegyptinone A (**3**), isolated from rhizomes of *Salvia tebesana* Bunge, had moderate cytotoxic activity against several cancer cell lines [29].

Compound **1**, with an IC_50_ value against BChE of 2.37 µM, also shows potential as a blocker of this target. Slightly less active was compound **2** with an IC_50_ value 7.86 µM. Interestingly, BChE is also supposed to play a role in the aggregation of Aβ (amyloid β) protein in the early stages of senile plaque formation in AD [32]. Those two compounds (**1**–**2**) have low anti-AChE activity in tested concentrations.

The selectivity of the studied ligands towards BChE versus AChE is probably caused by the ability to fit and utilize the additional space in the gorge of the active site.

The presence of active site residues lying at the bottom of a 20 Å deep hydrophobic gorge of BChE was revealed by its three-dimensional structure [32]. This information is highly relevant to the discovery of other new molecules in the same context. Structural analysis of docking results for the four studied compounds for which IC_50_ was calculated is displayed in Figure 2 and Figure 3, which show a representation of the active site of the enzyme with the docked compounds having highlighted hydrogen bonds. The so-called dual binding site inhibitors—compounds that can interact with the catalytic site (CAS) and peripheral anionic site (PAS) of the enzyme—are potential inhibitors of both ChE activity and Aβ aggregation [33,34]. In the investigated detailed representation of docking poses, we could observe the directionality of the network of established hydrogens bond. The residues from the catalytic triad (yellow color) and peripheral anionic site (green color) are involved in the formation of main hydrogen bonds with the studied compounds. Therefore, studied compounds block access to key residues involved in the normal functioning of the enzyme such as parts of the catalytic triad and PASs. Moreover, the interactions of isograndifoliol (**4**) with BChE result from the significant contribution of hydrophobic residues (Figure 3D), while the ligand is additionally stabilized by directional hydrogen bonds with Ser198 and His438 as in the case of 1–3 (Figure 3A–C). This kind of interaction can explain the markedly stronger inhibition of BChE by isograndifoliol and relatively weaker binding of (15*R*)-1-oxoaegyptinone A (**3**). The significance of hydrophobic residues in stabilizing the ligand within the BChE cavity was also observed for hyperforin and several prenylated hop flavonoids [33,34].

Using the ProTox-II prediction, it is evident that all four compounds are relatively safe in terms of their toxicity profiles. Compounds **1** and **3** belong to acute toxicity class 3 (toxic if swallowed, with LD_50_ values less than 300 mg/kg) and compounds **2** and **4** belong to acute toxicity class 4 (harmful if swallowed, with LD_50_ values less than 2000 mg/kg). Additionally, compounds **1**, **2**, and **3** have been predicted to be strongly immunotoxic. Compound **4** has shown 73.25% and 72.37% average similarity to the known ligands of toxicity targets glucocorticoid receptor and progesterone receptor, respectively. Compound **2** is predicted to be an inhibitor of CYP2C9 and compound **4** is predicted to be an inhibitor of CYP3A4 and CYP2C9 (as shown in Table 3). 

Using the admetSAR server, all four compounds were predicted as positive for the blood–brain barrier as well as positive for human intestinal absorption with very strong probabilities. The water solubility and the lipophilicity of all four compounds are predicted within the respective desired ranges (as shown in Table 4). Nevertheless, the in silico data require further verification using living models, such as cell lines, invertebrate, and vertebrate models. The theoretically predicted properties are therefore important for directing future experiments.

Moreover, the calculated affinity of arucadiol to the glucocorticoid receptor (GR) implies a second potential target for Alzheimer’s disease (AD) prevention to be verified using cellular or in vivo models. GRs are postulated to play a central role in the AD [35]. In addition, an apparent structural similarity to the steroid scaffold suggests a need to verify its potential as a neuroinflammation inhibitor.

For such experimental approach, inspired by the in silico study, much higher amounts of a preselected bioactive compound have to be isolated.

## 3. Materials and Methods 

### 3.1. General Experimental Procedures 

NMR data were acquired on a Bruker Avance III 500 MHz spectrometer (Bruker, BioSpin, Rheinstetten, Germany) equipped with 5 mm **^1^**H{**^109^**Ag–**^31^**P} broadband inverse (BBI) probe operating at room temperature at 500.20 MHz for **^1^**H, and 125.78 MHz for **^13^**C experiments. Structure assignment was achieved by a comprehensive analysis of 1D (**^1^**H, **^13^**C, selective-TOCSY) and 2D NMR data (**^1^**H-**^1^**H COSY, **^1^**H-**^1^**H ROESY, **^1^**H-**^13^**C H2BC, HMBC, and HSQC) and by comparison with literature data. The isolated substances’ purity was calculated by integration of the **^1^**H NMR spectra and exceeded 95%. For data processing, Topspin 3.5pl2 software (Bruker, Rheinstetten, Germany) was used. Samples were dissolved in 0.2 mL of deuterated methanol (MeOH-*d*_4_) and analyzed at 25 °C. 

Molecular formulas, exact masses, and MS/MS fragmentation were determined using a high-resolution quadrupole time-of-flight mass spectrometer (HRQTOF-MS, Impact II HD, Bruker Daltonik GmbH, Bremen, Germany). The ion source was operated in the negative and positive electrospray ionization modes using capillary voltage at 3.0 and 4.5 kV, respectively; nitrogen as nebulizer and drying gas at 2.0 bar and 6.0 L min^−1^, respectively; drying temperature of 200 °C; and mass scan range set from *m*/*z* 50 to *m*/*z* 2000. The instrument was calibrated for accurate mass measurement with a 10 mM sodium formate cluster applied via a 20 μL loop between each analysis. The MS/MS spectra were acquired using variable collision energy from 15 to 45 eV. Data were collected and processed by the Data Analysis 4.3 software (Bruker Daltonik GmbH, Bremen, Germany). 

Isolation of individual compounds was accomplished using a semi-preparative HPLC system equipped with a Gilson 321 pump, a Gilson GX-271 liquid handler with a 2 mL sample loop, a Gilson Prep ELS II detector (Gilson, Villiers le Bel, France), and the semi-preparative reversed-phase column Atlantis Prep T3 250 mm × 10 mm i.d., 5 μm (Waters, Milford, MA). The fractions were dried using a Gamma 2–16 LSC freeze dryer (Martin Christ Gefriertrocknungsanlagen GmbH, Osterode am Harz, Germany). 

For purification procedure checking and qualification of obtained fractions, we used a Waters ACQUITY UPLC system (Waters Corp., Milford, MA, USA), consisting of a sample manager, binary pump system, column manager, and PDA detector. The following analytical column was used: HSS Waters C18, dimensions 100 mm × 2.1 mm i.d., 1.7 μm, with VanGuard Pre-column (HSS 5 mm × 2.1 mm i.d., 1.8 μm) maintained at 40 °C. The eluent consisted of mobile phase A (0.1% FA in Milli-Q water, *v*/*v*) and mobile phase B (0.1% FA in MeCN, *v*/*v*). The gradient program was as follows: 0–0.5 min, 1% B; 0.5–10.0 min, 1–50% B; 10.0–11.0 min, 50–99% B; 11.0–13.0 min, 99% B; 13.0–13.05 min, 1% B; 13.05–15.0 min, 1% B. The flow rate was adjusted to 0.4 mL·min^−1^. Samples were kept at 8 °C in the sample manager. The injection volume of the sample was 3.0 μL (full loop mode) and samples were analyzed in triplicate. For needle washing, a strong solution (1:1:1, MeCN-MeOH-*i*PrOH, *v*/*v*/*v*) and a weak solution (5:95, MeCN–H_2_O, *v*/*v*) were used. The scan wavelength was set at 200-500 nm at a 5-point s^−1^ rate, with 2.0 nm resolution. Peaks were assigned according to their UV spectra, mass to charge ratios (*m*/*z*), and ESI-MS/MS fragmentation patterns. The MS analyses were carried out on a TQD mass spectrometer (Waters Corp.) equipped with a Z-spray electrospray interface. The following instrumental parameters were used for ESI-MS in positive ionization mode: capillary voltage, 3.0 kV; cone voltage, 40 V; desolvation gas, N_2_ 800 L h^−1^; cone gas, N_2_ 100 L h^−1^; source temperature 140 °C, and desolvation temperature 350 °C. Compounds were analyzed in full scan mode (mass range of *m*/*z* 100–1600). The stereochemistry of isolated compounds was additionally verified by circular dichroism (CD) spectroscopy and comparison of measured and calculated spectra (see Supplementary materials for details).

The concentrations of the four compounds were calculated from plotted calibration curves based on six concentration points (from 500 to 6 µg·mL^−1^) and recalculated to the dry mass of *P. atriplicifolia* roots. All analyses were performed in triplicate. 

### 3.2. Chemicals

All solvents used in this study—acetonitrile, dichloromethane, ethyl acetate, methanol, *n*-hexane—were of analytical grade and purchased from Merck (Darmstadt, Germany). The deuterated solvents—water-D2, methanol-D4, and 3-(trimethylsilyl)-1-propanesulfonic acid sodium salt (TSP)—were purchased from Armar AG (Döttingen, Switzerland) and ultrapure water was produced using the Milli-Q Simplicity 185 system (Millipore Corp., Billerica, MA, USA). Formic acid (FA), MS-grade, was purchased from Sigma-Aldrich (St. Louis, MO, USA). The LiChroprep RP-18, Silica gel 60 (60–200 mesh) was purchased from Merck-Sigma-Aldrich (Steinheim, Germany).

### 3.3. Plant Material

The root samples of cultivated *P. atriplicifolia* ‘Blue Spire’ were harvested in the fall of 2016 from the Botanical Garden of Medicinal Plants in Wrocław, Poland (17°04′27″ E, 51°07′03″ N, altitude 117 m asl). The collection is certified under the approval of the Ministry of Environment, Republic of Poland, Decision No. DOPogiz-4210-26-6024-/05/kL. The voucher specimens are kept in the herbarium of the Botanical Garden under the accession no. “Lamiaceae-Patriplicifolia 2016/1-4”. 

### 3.4. Isolation Procedure of Compounds ***1**–**4***

The isolation procedure of compounds **1**–**4** from *P. atriplicifolia* was a continuation of previous research [17,19]. The dried and ground well-developed roots (of ca. 1–2 cm in diameter) of *P. atriplicifolia* (250 g) were extracted in an ultrasonic bath (IS-40, Intersonic, Poland) with *n*-hexane (1.4 L) four times at 25 °C for 4 h each. The solvent was evaporated in vacuo and the obtained extract was lyophilized, which finally yielded 2.87 g of dried *n*-hexane extract. Afterward, the extract was applied onto a glass column filled with silica gel (400 g, 60 × 5 cm). The column was eluted with a dichloromethane-ethyl acetate step gradient with EtOAc increasing from 5% to 100%. As a result, 16 fractions were obtained and combined according to layer chromatography (TLC, Merck SG-60 F_254_) and checked by UPLC-PDA-MS. Fractions 1–13 were analyzed and described in detail previously [19]. For further separation, fractions (F14–16) were applied separately on a glass column filled with LiChroprep RP-18 (40–63 µm), and eluted isocratically as follows: F14- MeOH:H_2_O:FA (6:4:0.1, *v*/*v*/*v*), to obtain compound **2** (63 mg); F15 MeOH:H_2_O:FA (7:3:0.1, *v*/*v*/*v*), to obtain compound **1** (2 mg), compound **3** (4.2 mg) and compound **4** (6 mg). Further purification was performed by means of semipreparative isocratic HPLC using MeOH:H_2_O:FA (8:2:0.1, *v*/*v*/*v*), at 4 mL·min^−1^, on Atlantis Prep T3 at 25 °C.

### 3.5. Identification of Compounds ***1**–**4***

All isolated compounds from *P. atriplicifolia* were identified based on MS/MS and NMR spectra and have been characterized as shown below. We obtained three new natural products (**1**–**3**):

(1*R*,15*R*)-1-Acetoxycryptotanshinone (**1**), red amorphous powder; UV λ_max_ (nm): 225, 270, 441, $\alpha_{D}^{20}$ +133 (*c* 0.056 CHCl_3_), HRQTOF-MS (neg.) *m*/*z* 353.1392 [M–H]- (calc. for C_21_H_22_O_5_− = 353.1394); positive mode, *m*/*z* 355.1538 [M+H]^+^ (calc. for = C_21_H_22_O_5_^+^ = 355.1540), HR-MS/MS (% of base peak) diagnostic fragment ions at *m*/*z* 295.1327(100) [M+H- 59]^+^ (calc. for C_19_H_19_O_3_^+^ = 295.1329), 249.1273(15), 277.1222 (8), 267.1381(3). For **^1^**H and **^13^**C NMR data, see Table 1.

(1*R*)-1-Acetoxytanshinone IIA (**2**), red needle-shaped crystals. UV λ_max_ (nm): 221, 250, 271, 460. $\alpha_{D}^{20}$ +203 (*c* 0.046 CHCl_3_), HRQTOF-MS (neg.) *m*/*z* 351.1236 [M-H]- (calc. for C_21_H_19_O_5_- = 351.1238), positive mode *m*/*z* 353.1385 [M+H]^+^ (calc. for C_21_H_21_O_5_^+^ = 353.1384, HR-MS/MS (% of base peak) diagnostic fragment ions at m/z 293.1172 (100) [M+H- 59]^+^ (calc. for C_19_H_17_O_3_ = 293.1178), 265.1229 (15).

(15*R*)-1-oxoaegyptinone A ((*R*)-3,10,10-trimethyl-2,3,9,10-tetrahydroanthra[1,2-*b*]furan-4,5,7(8*H*)-trione) (**3**), red needle-shaped crystals. UV λ_max_ (nm): 224, 240, 290, 317, $\alpha_{D}^{20}$ −3.75 (*c* 0.06 CHCl_3_), HRQTOF-MS (neg.) *m*/*z* 309.1236 [M-H]- (calc. for C_19_H_17_O_4_- = 309.1238), positive mode *m*/*z* 311.1277 [M+H]^+^ (calc. for C_19_H_19_O_4_^+^ = 311.1278, HR-MS/MS (% of base peak) diagnostic fragment ions at *m*/*z* 283.1319 (40) [M+H- 28]^+^ (calc. for C_18_H_19_O_3_^+^ = 283.1327), 265.1220 (60).

Isograndifoliol (**4**), brownish amorphous powder. UV λ_max_ (nm): 246, 320, 365, $\alpha_{D}^{20}$ +51.3 (*c* 0.06 CHCl_3_), HRQTOF-MS (neg.) *m*/*z* 301.1802 [M-H]- (calc. for C_19_H_25_O_3_- = 301.1809), positive mode *m*/*z* 303.1953 [M+H]^+^ (calc. for C_19_H_27_O_3_^+^ = 303.1955, HR-MS/MS (% of base peak) diagnostic fragment ions at *m*/*z* 271.1685 [M-H-30]^−^ (calc. for C_18_H_23_O_2_- = 271.1698 (40), 243.1748 [M-H-30-28]- (calc. for C_17_H_23_O- = 243.1754 (100).

### 3.6. Microtiter Assays for AChE and BChE Enzyme Inhibition

A modified spectrophotometric method of Ellman et al. [36] was used for the determination of inhibitory activity against AChE and BChE. Electric eel acetylcholinesterase (Type-VI-S, EC 3.1.1.7, Sigma, St. Louis, MO, USA) and horse serum butyrylcholinesterase (EC 3.1.1.8, Sigma, St. Louis, MO, USA) were used as the test enzymes and acetylthiocholine iodide and butyrylthiocholine chloride (Sigma, St. Louis, MO, USA) were employed as the respective substrates. The ChE activity was evaluated with the detection reagent 5,5′-dithio-bis(2-nitrobenzoic) acid (DTNB, Sigma, St. Louis, MO, USA). All the other reagents and conditions were the same as described in our previous publication [37]. In brief, the following reaction mixture was added by a multichannel automatic pipette (Gilson Pipetman, Villiers Le Bel, France) in a 96-well microplate and incubated for 15 min at 25 °C: 140 µL of 0.1 mM sodium phosphate buffer (pH 8.0), 20 µL of 0.2 M DTNB, 20 µL of sample solutions (0.1–10 µg/mL) and 20 µL of acetylcholinesterase/butyrylcholinesterase solution (0.2 µM). The reaction was then initiated with the addition of 10 µL of acetylthiocholine iodide or butyrylthiocholine chloride (2 mM). The hydrolysis of acetylthiocholine and butyrylthiocholine was monitored by the formation of the yellow 5-thio-2-nitrobenzoate anion at 412 nm using the 96-well VersaMax (Molecular Devices, San Jose, CA, USA) microplate reader. Galanthamine (Sigma, St. Louis, MO, USA)—an alkaloid isolated from snowdrop—was employed as the reference inhibitory drug.

### 3.7. Data Processing for Enzyme Inhibition Assays

The measurements and calculations were performed using SoftMax PRO 4.3.2.LS software (Molecular Devices, San Jose, CA, USA). The percentage of inhibition of AChE/BChE was determined by comparison of rates of reaction of test samples relative to a blank sample (ethanol in phosphate buffer, pH = 8). The extent of the enzymatic reaction was calculated based on the following equation: *E* = (*C* − *T*)/*C* × 100, where *E* is the activity of the enzyme. *E* expresses the effect of the test sample or the positive control on acetylcholinesterase and butyrylcholinesterase enzyme activity expressed as the percentage of the remaining activity in the presence of the test sample or positive control. *C* is the absorbance of the control solvent (blank) in the presence of an enzyme, and *T* is the absorbance of the tested sample (plant extract or positive control in the solvent) in the presence of an enzyme.

### 3.8. Statistical Analysis of Data

Data are expressed as average inhibition ± standard error of the mean (S.E.M.) and the results were taken from at least three independent experiments performed in triplicate. Statistical differences between the reference and the sample groups were evaluated by one-way ANOVA (Statistica 13PL, StatSoft, Cracow, Poland) with Dunnett’s multiple comparison post-hoc test for which *p* < 0.05 was considered to be significant (* *p* < 0.05; ** *p* < 0.01; *** *p* < 0.001, **** *p* < 0.0001).

### 3.9. Molecular Modeling

To build up the initial geometries of the norditerpenoids, GaussView was used to set to the standard parameters, followed by full optimization at the B3LYP/6-31G(d) level within the density functional theory (DFT) framework [38,39]. The stability of all localized structures was confirmed by performing additional vibrational calculations, e.g., all minima in the potential energy surface present real frequencies only. The DFT study was finally completed as described in the Merz-Singh-Kollman ESP protocol by computing all partial atomic charges [40,41]. All DFT calculations were performed with the Gaussian 16 suite program [42]. The molecular structures for the AChE and BChE enzymes were extracted from the crystal structures of Protein Data Bank (PDB) files with codes 1EVE and 1P0I, respectively. Their molecular files were prepared with Autodock Tools (The Scripps Research Institute, La Jolla, CA, USA) for docking by removing water molecules, adding hydrogen atoms and Gasteiger charges, merging non-polar hydrogens and assigning AD4 atom types [43]. Molecular docking calculations were carried out in the active site of both enzymes using default parameters in AutoDock Vina [43]. Graphical representations of the docking results were prepared in 2D using Poseview and in 3D using PyMOL (Molecular Graphics System, version 1.6, Schrödinger LLC, New York, NY, USA) [44].

### 3.10. Computational Prediction of ADMET (Absorption, Distribution, Metabolism, Excretion, and Toxicity) Properties

To understand the potential toxic as well as drug-likeness profiles of the four compounds, computational toxicity prediction analysis was performed using ProTox-II (a freely available virtual platform for the prediction of chemical-based toxicities) and admetSAR servers, respectively [45,46,47]. Currently, the ProTox platform includes 33 models for the prediction of several endpoints like acute toxicity, organ toxicity, off-target toxicity, carcinogenicity, cytotoxicity, immunotoxicity, and mutagenicity, as well as adverse outcome pathways. Similarly, admetSAR is a freely available tool that helps in evaluating several multiple toxicity endpoints, drug-likeness and metabolism of small molecules. For the prediction of cytochrome activity of the four compounds, in-house models based on the SuperCyp database were used in this study [48].

## 4. Conclusions

In conclusion, screening of diterpenoids from *P. atriplicifolia* roots for their cholinesterase inhibitory potential resulted in the discovery of a set of four novel selective BChE inhibitors. The molecular modeling results enlighten the main interactions involved between the compounds and the enzyme. Two of the isolated compounds, (1*R*,15*R*)-1-acetoxycryptotanshinone (**1**) and isograndifoliol (**4**), seem to be quite promising natural compounds that can serve as a lead structure for pharmacological dementia management. Compounds **1**, **2**, and **3** are new natural products that can be quite easily isolated from the easy-to-grow plant. The presence of significant amounts of isograndifoliol suggests that *P. atriplicifolia* could be a new major natural source of this rarely reported compound.

## Figures and Tables

**Table 1:** **^1^**H (500 MHz) and **^13^**C (125 MHz) NMR data of 1–3 in methanol-*d*_4_ (δ in ppm, *J* in Hz) *.

Position	1		2		3	
	δ_C_ type	δ_H_ (*J* in Hz)	δ_C_ type	δ_H_ (*J* in Hz)	δ_C_ type	δ_H_ (*J* in Hz)
1	68.7, CH	6.40, t (3.4)	69.0, CH	6.41, t (3.4)	201.8, C	
2	25.5, CH_2_	2.13–2.19, m 2.02, tdd (14.0, 3.5, 2.5)	25.6, CH_2_	2.13–2.19, m 2.01, tt (14.1, 2.6)	37.3, CH_2_	2.90, t (7.2)
3	33.1, CH_2_	1.93, td (13.3, 2.6) 1.59, ddd (13.3, 5.5, 2.5)	33.2, CH_2_	1.93, td (13.1, 2.4) 1.58, ddd (12.9, 5.6, 2.8)	37.5, CH_2_	2.10, t (7.2)
4	35.9, C		35.8, C		36.4, C	
5	154.2, C		151.9, C		138.7, C	
6	134.9, CH	7.84, d (7.7)	135.7, CH	7.89, d (8.3)	157.9, C	
7	126.4, CH	7.67, d (7.8)	124.2, CH	7.80, d (8.3)	131.7, CH	7.78
8	128.3, C		129.6, C		128.6, C	
9	130.1, C		128.2, C		134.2, C	
10	138.1, C		138.7, C		128.2, CH	7.78
11	184.5, C		183.8, C		184.9, C	
12	176.3, C		176.3, C		178.9, C	
13	119.6, C		121.1, C		120.4, C	
14	172.7, C		162.7, C		172.0, C	
15	35.7, CH	3.48–3.58, m	122.3, C		35.8, CH	3.54–3.64, m
16	83.2, CH_2_	*α* 4.96, t (9.4) *β* 4.42, dd (9.3, 6.2)	143.6, CH	7.48, q (1.3)	83.6, CH_2_	*α* 5.01, t (9.6) *β* 4.48, dd (9.5, 6.2)
17	18.7, CH_3_	1.32, d (6.8)	8.8, CH_3_	2.23, d (1.2)	18.8, CH_3_	1.347, d (6.9)
18	31.3, CH_3_	1.28, s	31.2, CH_3_	1.31 s	28.9, CH_3_	1.352, s
19	31.9, CH_3_	1.39, s	31.9, CH_3_	1.41, s	28.9, CH_3_	1.350, s
1-*O*-**CO**-Me	171.8		172.2			
1-*O*-CO-**Me**	21.0	1.99, s	21.0	2.01, s		

* Overlapped signals are reported without designating multiplicity.

**Table 2:** Cholinesterase (AChE and BChE) inhibition by the four isolated compounds (**1**–**4**) and their content (mg g^−1^ D.W.) in roots of *Perovskia atriplicifolia* measured using Diode-Array Detector (DAD)-HPLC.

Compound Number	Compound	% Inhibition ± S.D. at 10.0 µg·mL^−1^		BChE Inhibition IC_50_		K_i_ app *	Content in Dried Roots
		AChE	BChE	µg·mL^−1^	µM	µM	mg (100 g)^−1^
**1**	**(1*R*,15*R*)-1-Acetoxycryptotanshinone**	22.8 ± 2.4	95.9 ± 0.0	0.84 ± 0.09	2.37	1.34	28.5 ± 2.5
**2**	**(1*R*)-1-Acetoxytanshinone IIA**	28.0 ± 0.9	85.3 ± 4.3	2.77 ± 0.48	7.86	4.59	8.1 ± 0.4
**3**	**(15*R*)-1-oxoaegyptinone A**	49.6 ± 1.8	87.3 ± 1.0	15.75 ± 1.12	50.80	30.0	21.3 ± 0.7
**4**	**Isograndifoliol**	50.0 ± 1.8	98.6 ± 0.0	0.27 ± 0.02	0.89	0.47	302.0 ± 9.1
REF **	**Galanthamine hydrobromide**	97.2 ± 2.9	86.8 ± 2.9	28.16 ± 1.51	76.4	---	--------------

* for assumed competitive inhibition, substrate concentration = 1 mM. ** reference drug.

**Table 3:** Results obtained from computational toxicity prediction using the ProTox-II platform.

Compound Name	Predicted LD_50_ Value and Tox class	Prediction Accuracy (%)	Toxicity Targets	Avg Similarity to Known Ligands (%)	Toxicity Endpoints	Prediction Probability	Cytochrome Inhibition Prediction
**1.** Acetoxycrypto-tanshinone	260 (mg/kg), Tox class: 3	54.26	-	-	Immunotoxicity	**0.99**	-
**2.** Acetoxytanshinone IIA	1230 (mg/kg), Tox class: 4	54.26	-	-	Immunotoxicity	**0.83**	CYP2C9 (62%)
**3.** 1-oxoaegyptinone A	260 (mg/kg), Tox class: 3	54.26	-	-	Immunotoxicity	**0.96**	-
**4.** Isograndifoliol	2000 (mg/kg) Tox class: 4	68.27	Glucocorticoid Receptor	73.25	-	-	CYP3A4 (78%)
			Progesterone Receptor	72.37			CYP2C9 (63%)

**Table 4:** Results obtained from computational prediction using the admetSAR platform.

Compounds	Blood-Brain Barrier	Probability	Water Solubility (logS)	Lipophilicity (AlogP)	Plasma Protein Binding (100 %)	Human Intestinal Absorption	Probability
**1.** Acetoxycryptotanshinone	positive	0.97	−4.43	3.51	0.89	positive	0.99
**2.** Acetoxytanshinone IIA	positive	0.93	−4.42	4.31	1.06	positive	0.98
**3.** oxoaegyptinone A	positive	0.96	−3.78	3.08	0.95	positive	0.99
**4**. Isograndifoliol	positive	0.93	−4.28	3.06	0.82	positive	0.99

## Supplementary Materials

Supplementary materials can be found at https://www.mdpi.com/1422-0067/21/12/4475/s1.

## Abbreviations

AChE	acetylcholinesterase
AD	Alzheimer’s disease
Aβ	amyloid plaques
BChE	butyrylcholinesterase
CAS	catalytic site
DFT	density functional theory
ECD	electronic circular dichroism
FA	Formic acid
GR	glucocorticoid receptor
HRQTOF-MS	high-resolution quadrupole time-of-flight mass spectrometer
MeOH-d4	deuterated methanol
PAS	peripheral anionic site
PDB	Protein Data Bank
