# Supplementary material for: Norditerpenoids with Selective Anti-Cholinesterase Activity from the Roots of Perovskia atriplicifolia Benth
Source: Int J Mol Sci. 2020 Jun 23;21(12):4475. doi: 10.3390/ijms21124475 (PMC7352922; doi:10.3390/ijms21124475)

SUPPLEMENTARY FILE

Norditerpenoids with Selective Anti-Cholinesterase Activity from the Roots of *Perovskia atriplicifolia* Benth.

Sylwester Ślusarczyk, F. Sezer Senol Deniz, Renata Abel, Łukasz Pecio, Horacio Pérez-Sánchez, José P. Cerón-Carrasco, Helena den-Haan, Priyanka Banerjee, Robert Preissner, Edward Krzyżak, Wiesław Oleszek, Ilkay Erdogan Orhan, and Adam Matkowski

CIRCULAR DICHROISM EXPERIMENT

Results

Experimental CD curves and calculated ECD spectra for all compounds are shown on Figure S1. In any case, the experimental curves show several positive and negative Cotton effects (CE). Strong maxima or minima occur at about 490, 265, and 220 nm for 1-acetoxycryptotanshinone; 500, 355, 270, 250, and 220 nm for acetoxytanshinone IIA; 370, 340, 295, and 220 nm for 1-oxoaegyptinone A; as well as 330, 285, 260, and 220 nm for isograndifoliol. These effects resulted from the π → π∗ transition in the aromatic part of studied compounds. The calculated spectra show patterns similar to those of the experimental spectra. Some differences may correspond to the differences in real conforming structure. However, based on these results, it can be concluded that the configuration of the studied compounds is as proposed based on NMR spectra analysis.

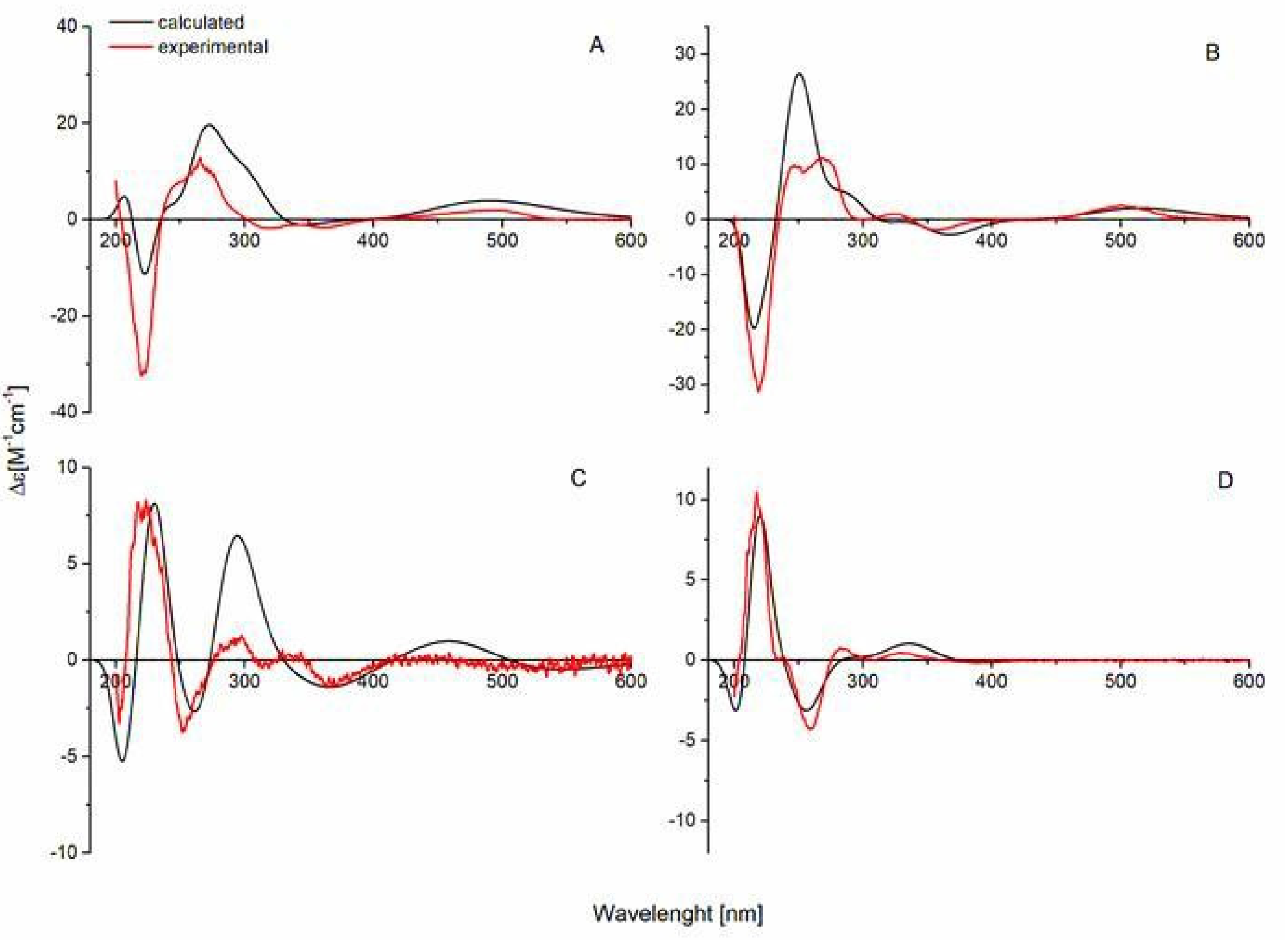

Supplementary Figure 1. Comparison of experimental and calculated CD spectra: **A**–1-(acetyloxy)-cryptotanshinone, **B**–acetoxytanshinone IIA, **C**–3,10,11-trimethyl-2,3,9,10-tetrahydroanthra[1,2,b]furan-4,5,7(8H)-trion, and **D**–isograndifoliol.

Experimental conditions

Circular dichroism

Circular dichroism spectra of all analyzed compounds were made in room temperature on a Jasco J-1500 magnetic circular dichroism spectrometer (JASCO, Japan). 1 mm pathlength was used. CD spectra were collected at a scan rate speed of 100 nm min^{-1} with response time of 1 s. Measurements were made in the range of 200–600 nm. All spectra were baseline corrected and the final plot was taken from three accumulated plots. The concentrations of compounds were from 0.25 mg/ml to 1.1 mg/ml.

Computational Methods

Calculations were performed using the Gaussian 2016 A.03 software package [1]. Conformation analysis of all compounds was performed using Monte Carlo. Conformers within a 2 Kcal/mol energy difference from the global minimum were selected for structure and electronic properties calculations. The ground state geometric optimizations were calculated using density functional theory (DFT) with Becke's three-parameter hybrid exchange function with the Lee–Yang–Parr gradient

corrected correlation (B3LYP) [2–4] functional in combination with 6–311+G (d,p) basis set. Harmonic

vibrational wavenumbers were calculated using analytic second derivatives to confirm the convergence to minima in the potential surface. The electronic properties, such as excitation energy and rotatory strengths, were calculated using time-dependent density functional theory at the TDDFT/6– 311+G (d, p) level. The hybrid functionals PBE0 [5] were employed in calculations. TDDFT

calculations also included solvent effect [6,7]. ECD curves were obtained on the basis of rotator strengths with a half-band of 0.25 eV using SpecDis v1.71 [8].

Acknowledgments

Calculations have been carried out in the Wroclaw Centre for Networking and Supercomputing (http://www.wcss.wroc.pl)

NMR SPECTRA OF ISOLATED COMPOUNDS

^{1}H (500 MHz) NMR spectrum of compound **1** in CD_3OD, 25°C

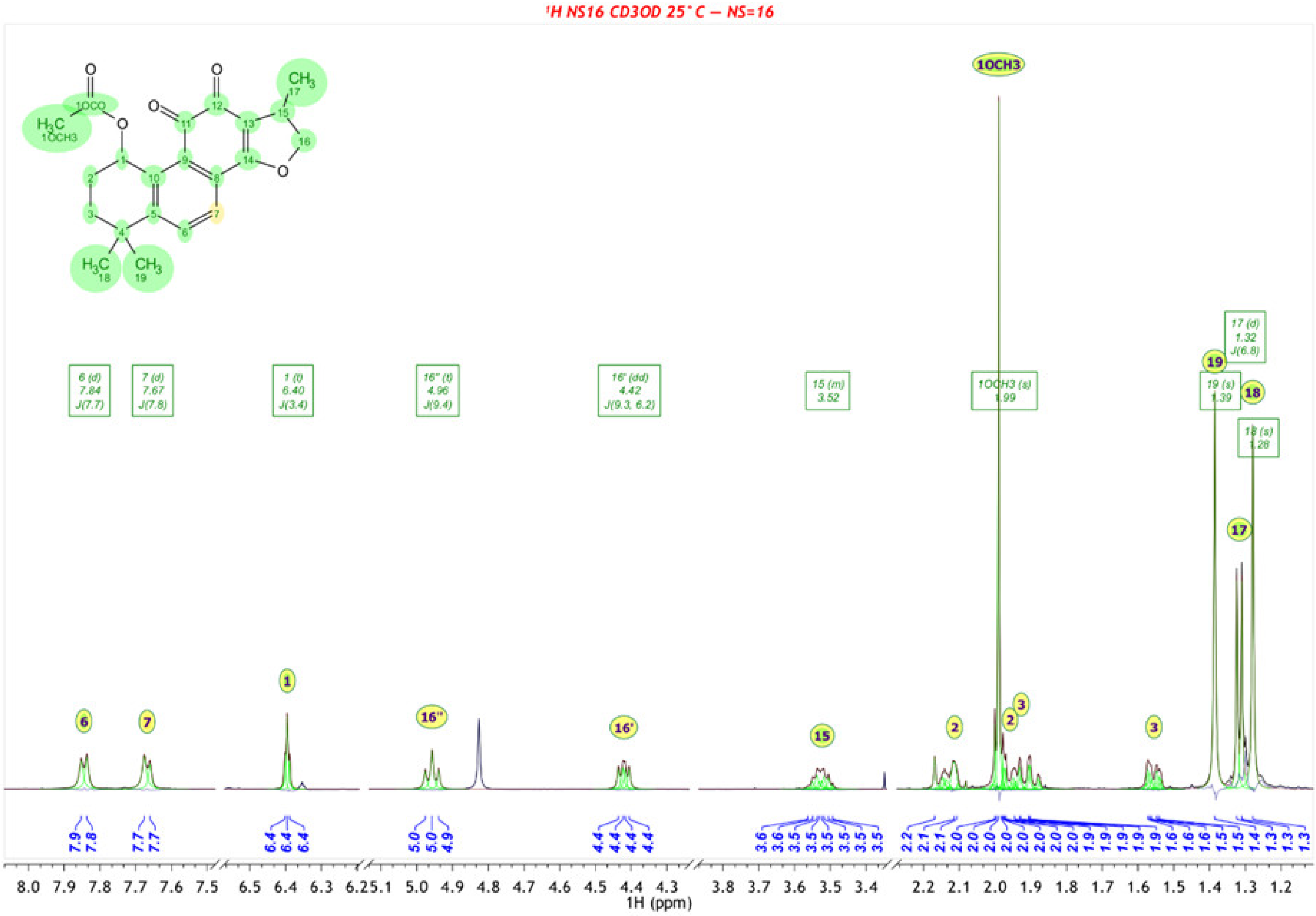

^{13}C (125 MHz) NMR spectrum of compound 1 in CD_3OD, 25°C

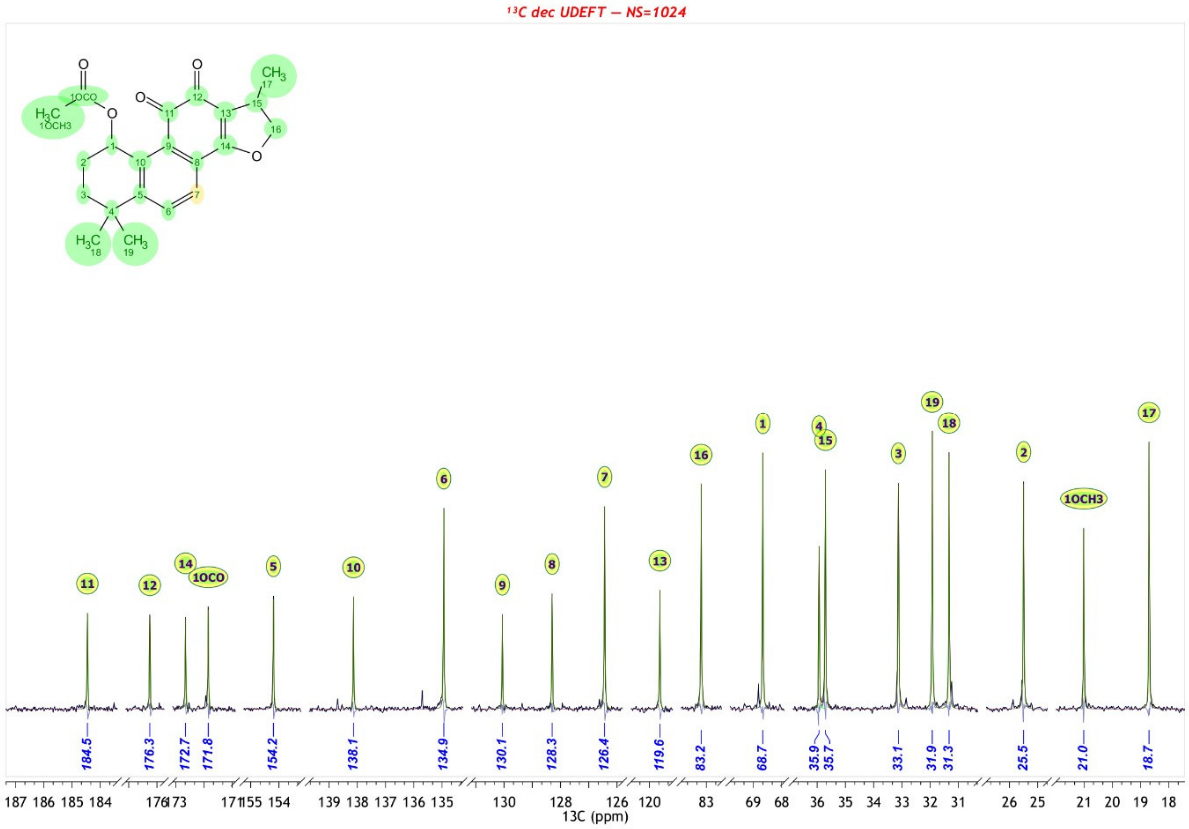

^1H-^1H COSY NMR spectrum of compound 1 in CD_3OD, 25°C

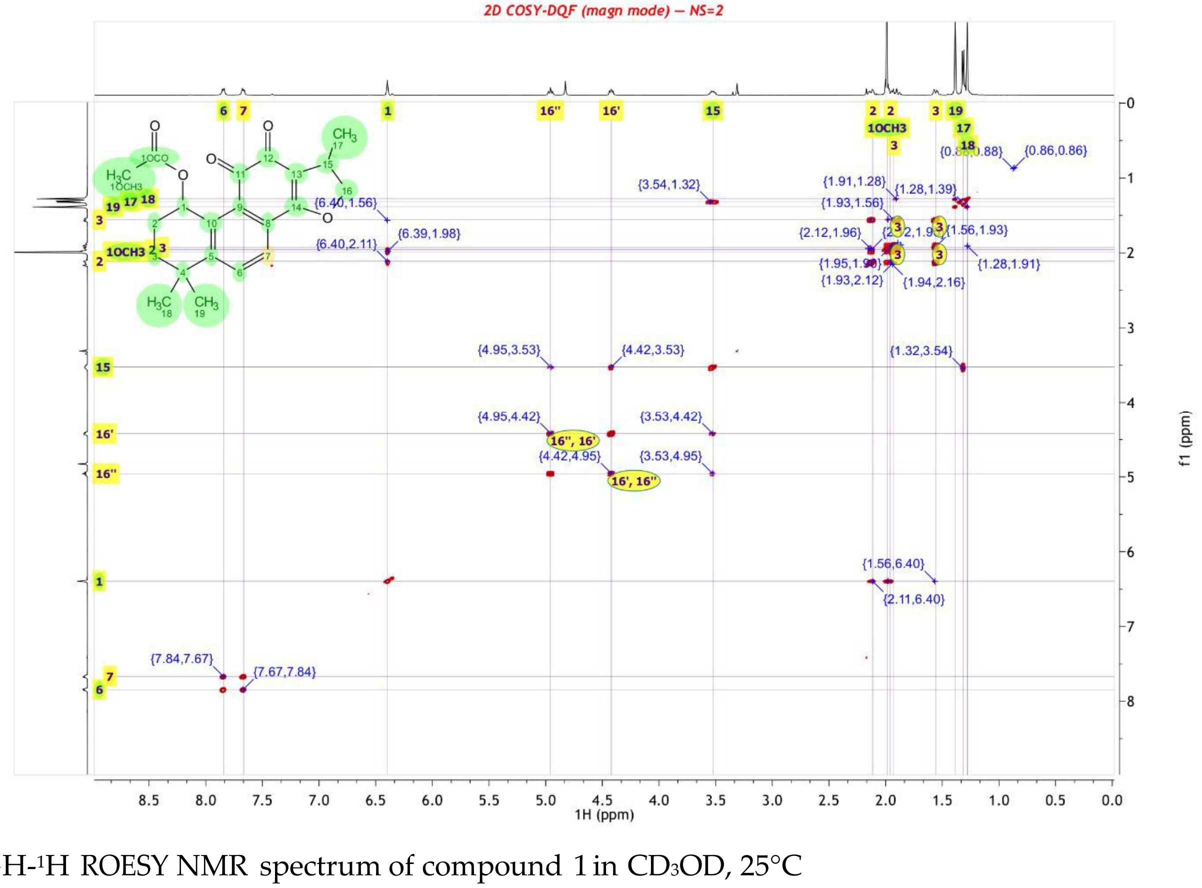

^1H-^1H ROESY NMR spectrum of compound 1 in CD_3OD, 25°C

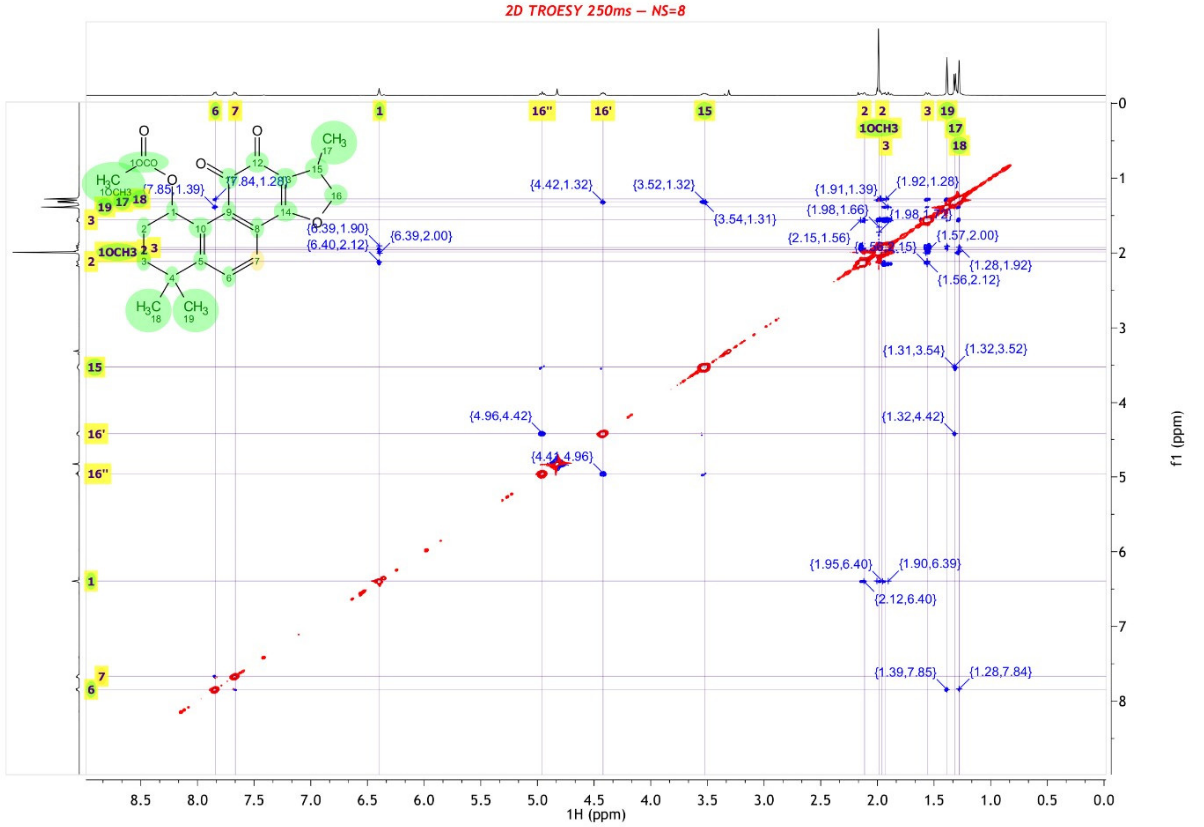

^{1}H-^{13}C HSQC NMR spectrum of compound 1 in CD_3OD, 25°C

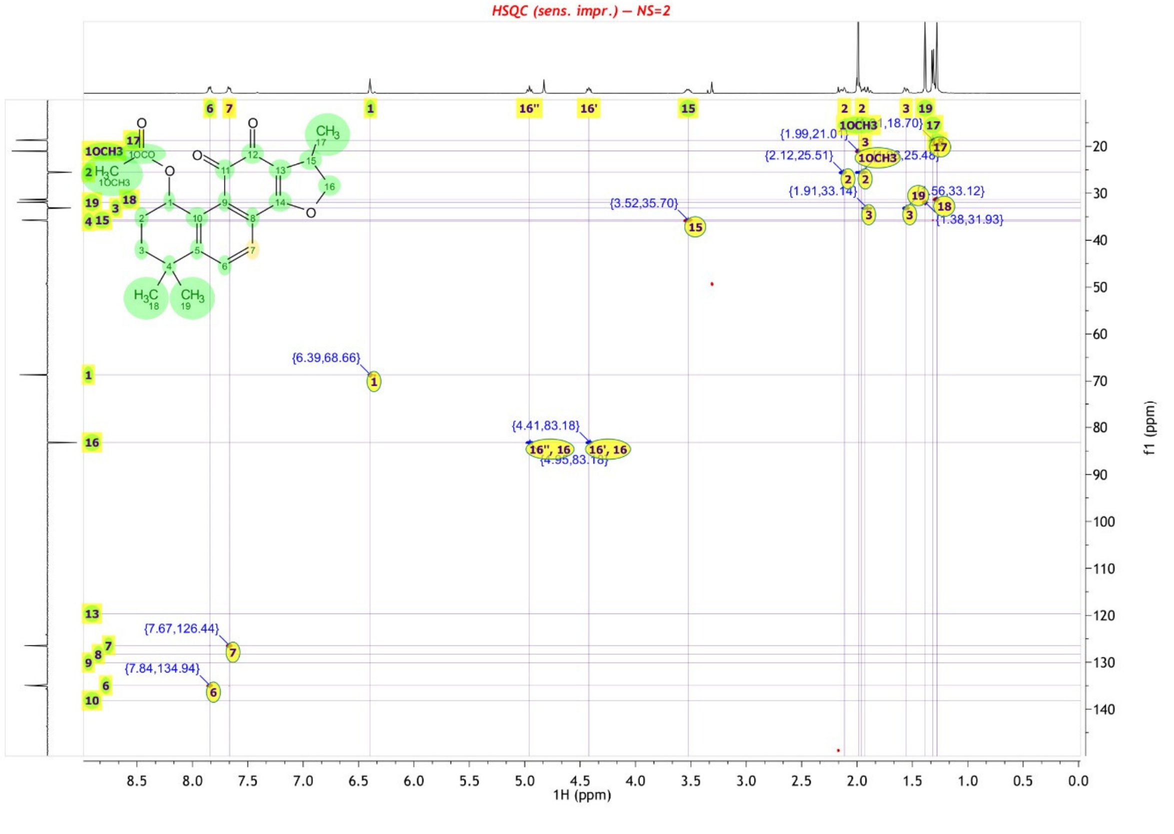

^{1}H-^{13}C H2BC NMR spectrum of compound 1 in CD_3OD, 25°C

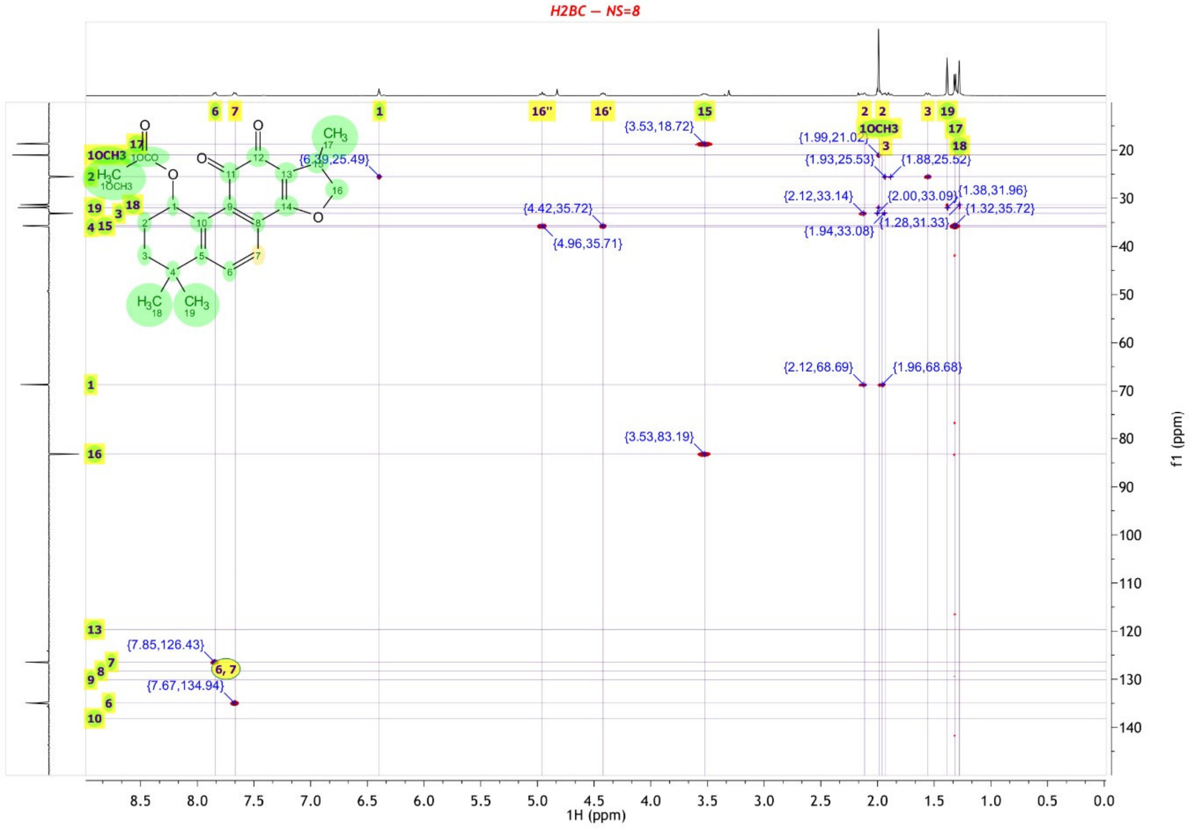

^1H-^{13}C HMBC 8 Hz NMR spectrum of compound 1 in CD_3OD, 25°C

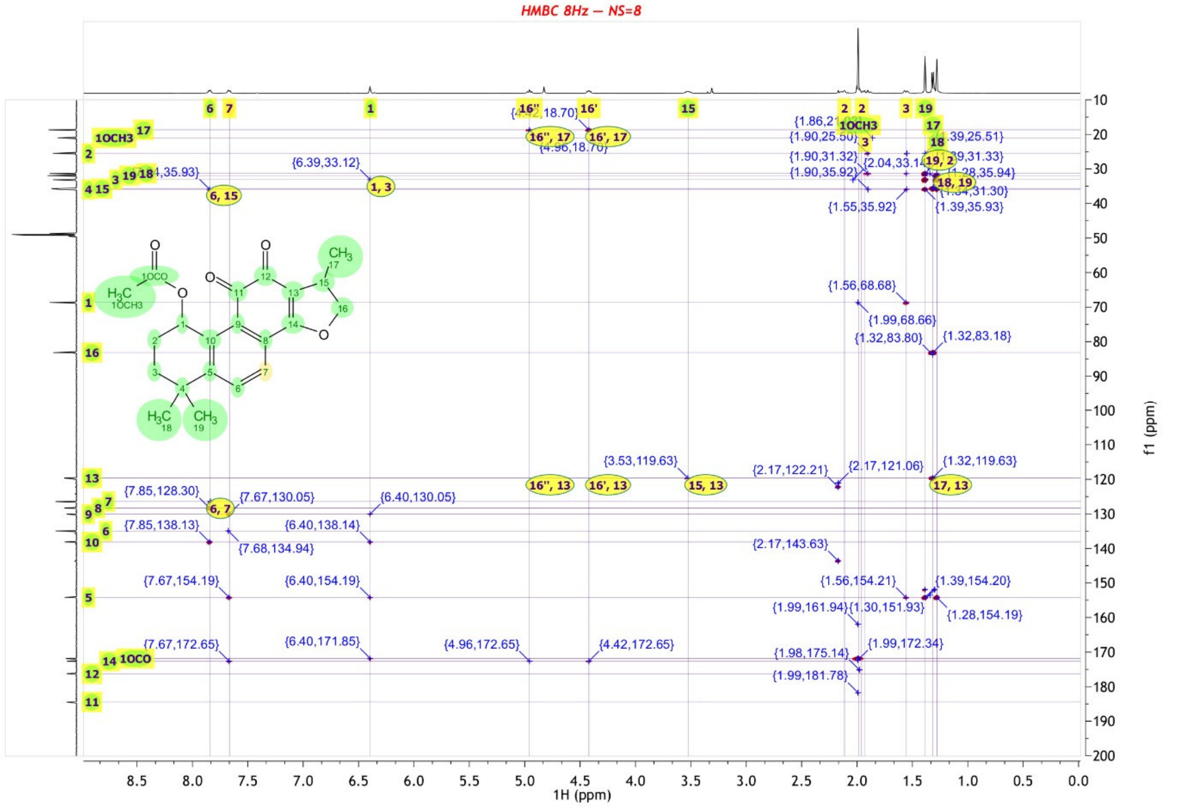

^1H-^{13}C HMBC 3 Hz NMR spectrum of compound 1 in CD_3OD, 25°C

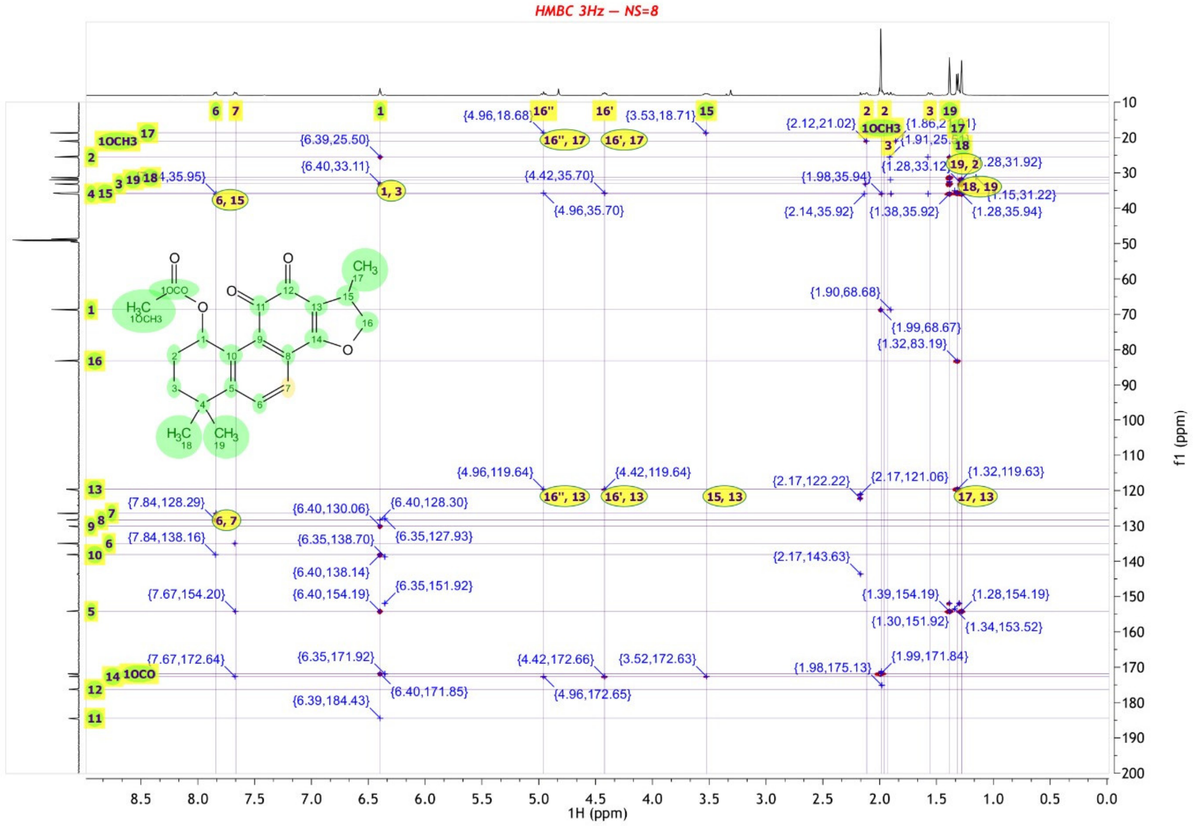

^{1}H (500 MHz) NMR spectrum of compound 2 in CD_3OD, 25°C

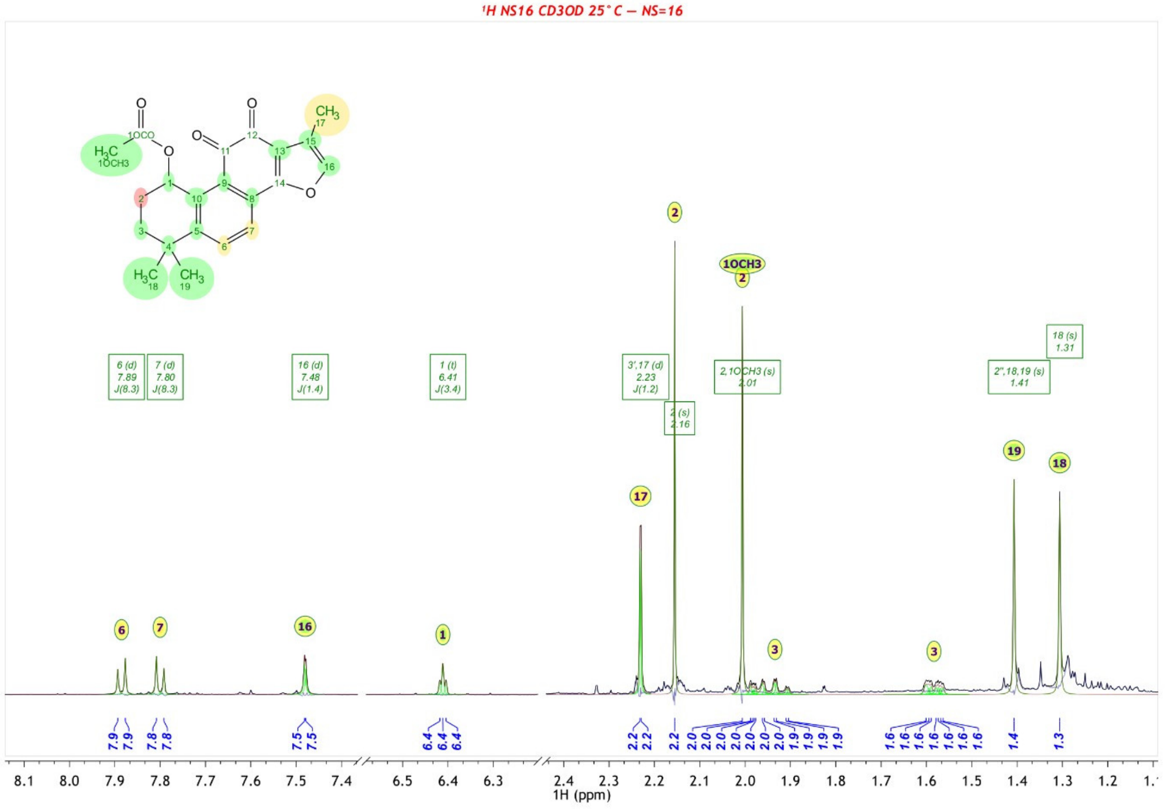

^{13}C (125 MHz) NMR spectrum of compound 2 in CD_3OD, 25°C

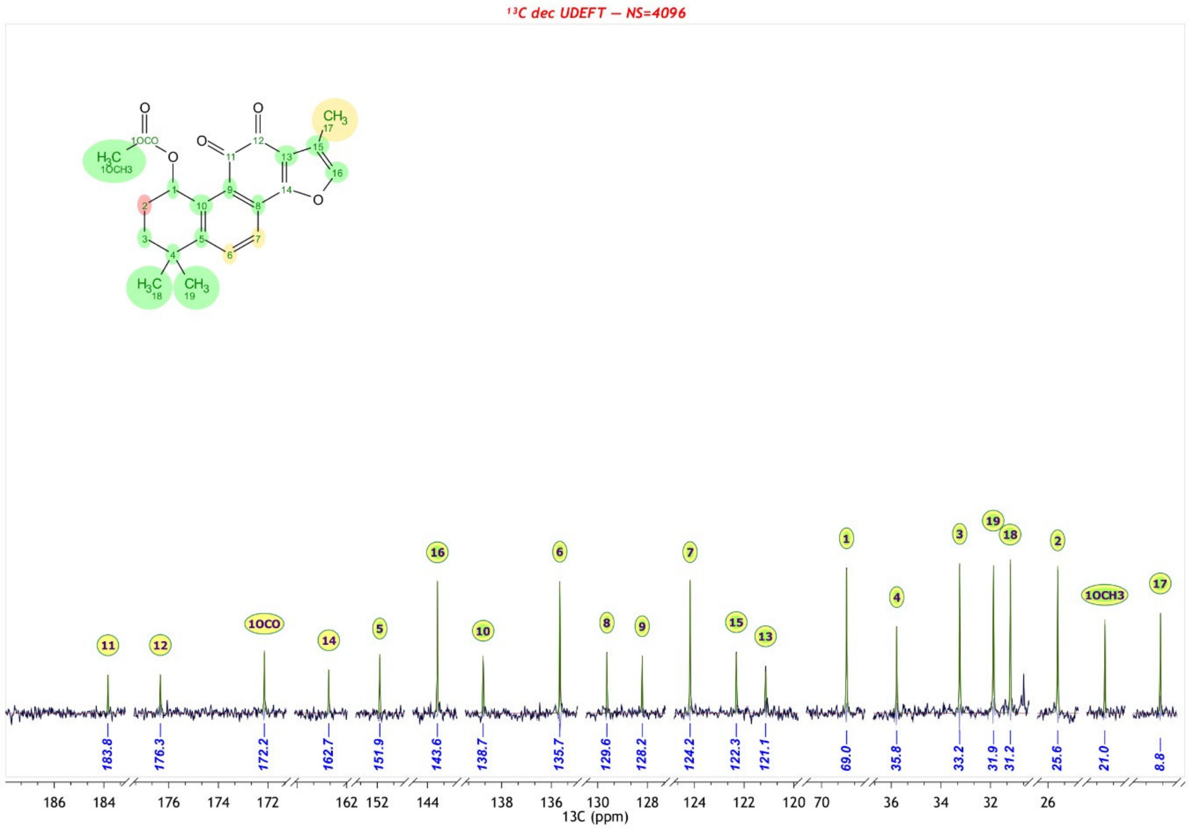

^1H-^1H COSY NMR spectrum of compound 2 in CD_3OD, 25°C

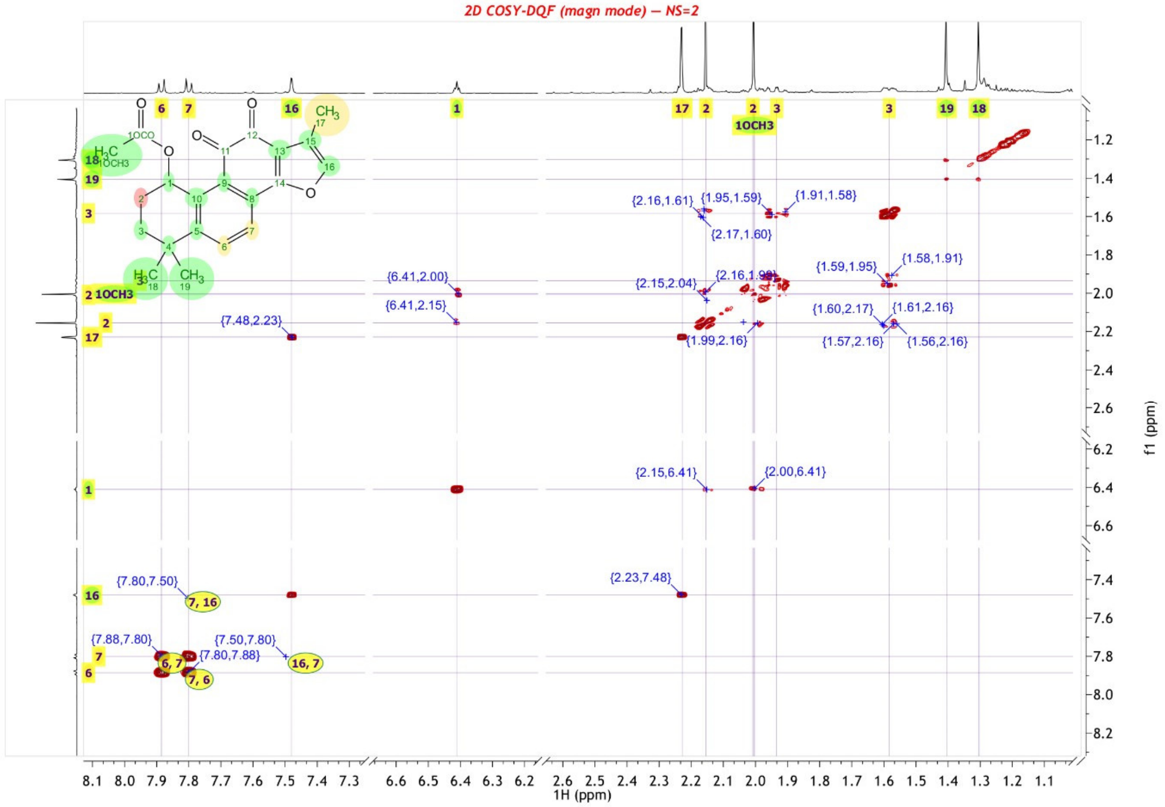

^1H-^1H TROESY NMR spectrum of compound 2 in CD_3OD, 25°C

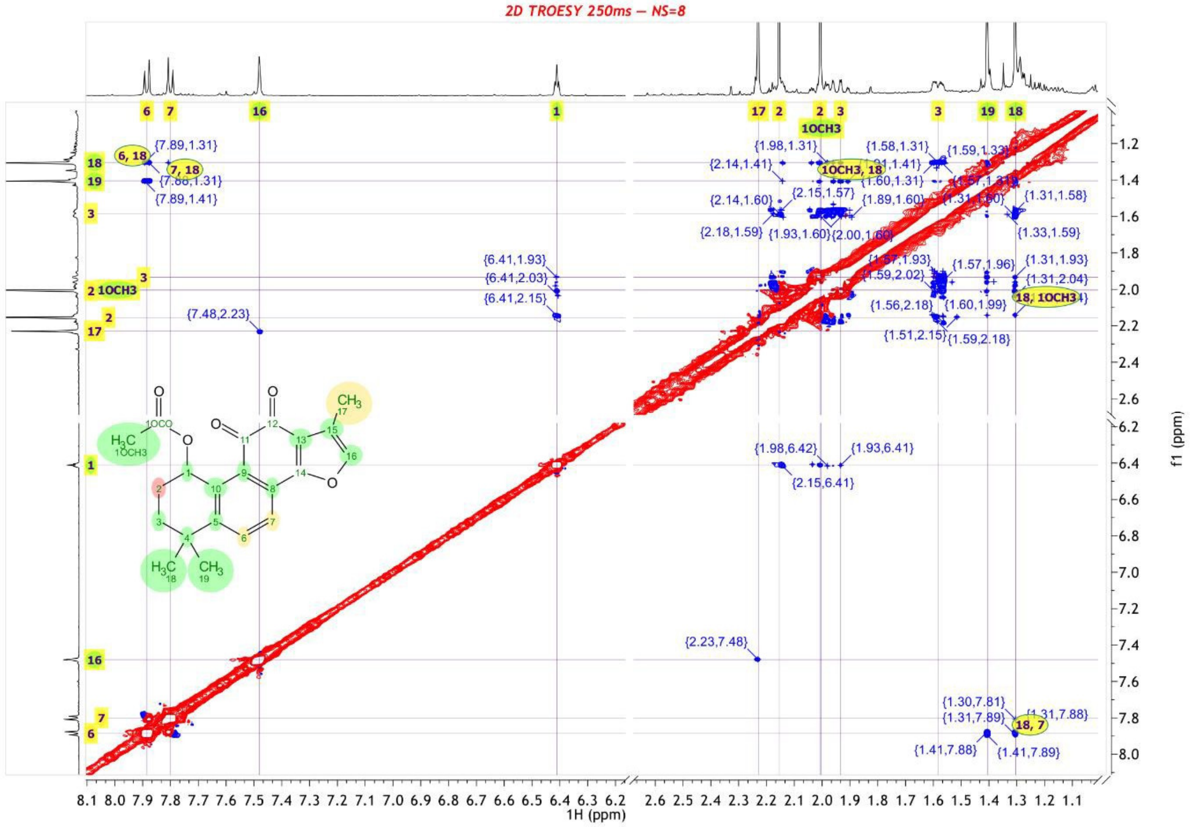

^{1}H-^{13}C HSQC NMR spectrum of compound 2 in CD_3OD, 25°C

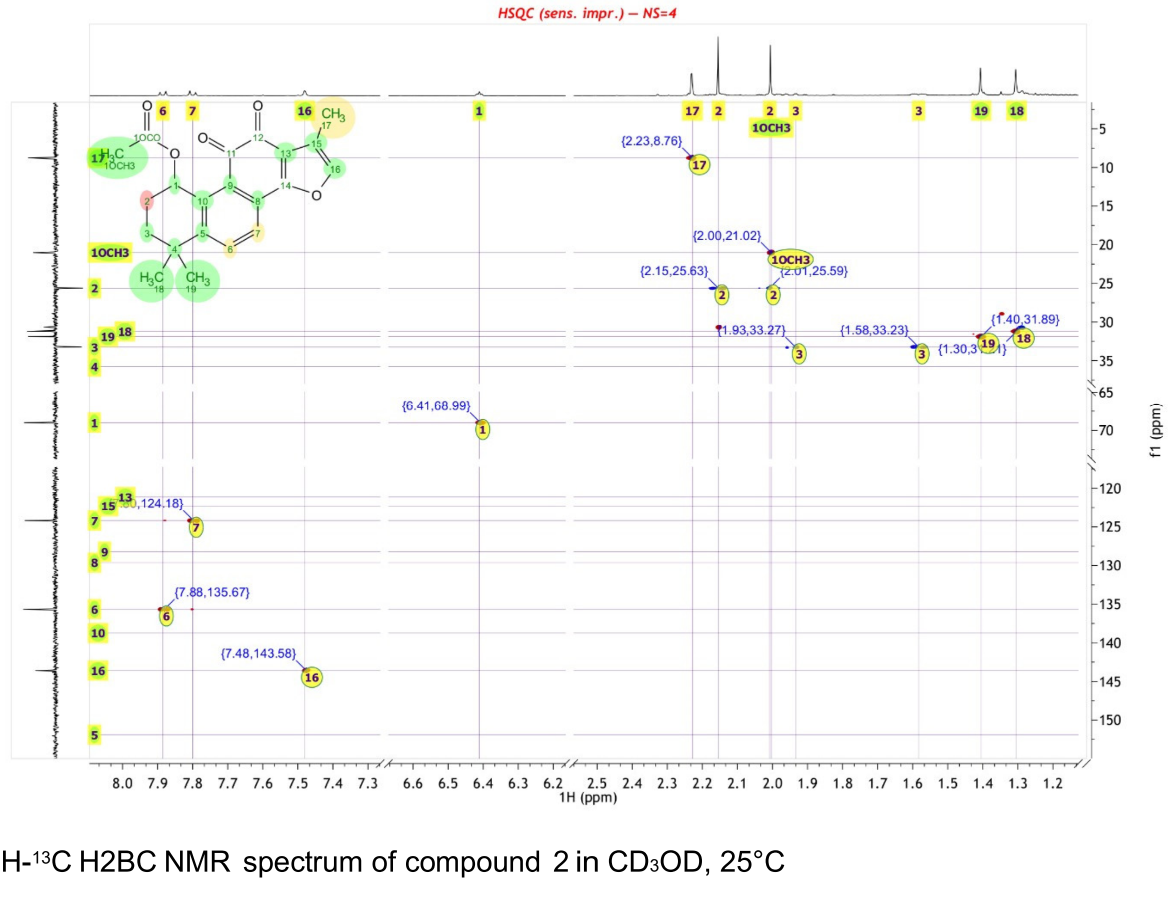

^{1}H-^{13}C H2BC NMR spectrum of compound 2 in CD_3OD, 25°C

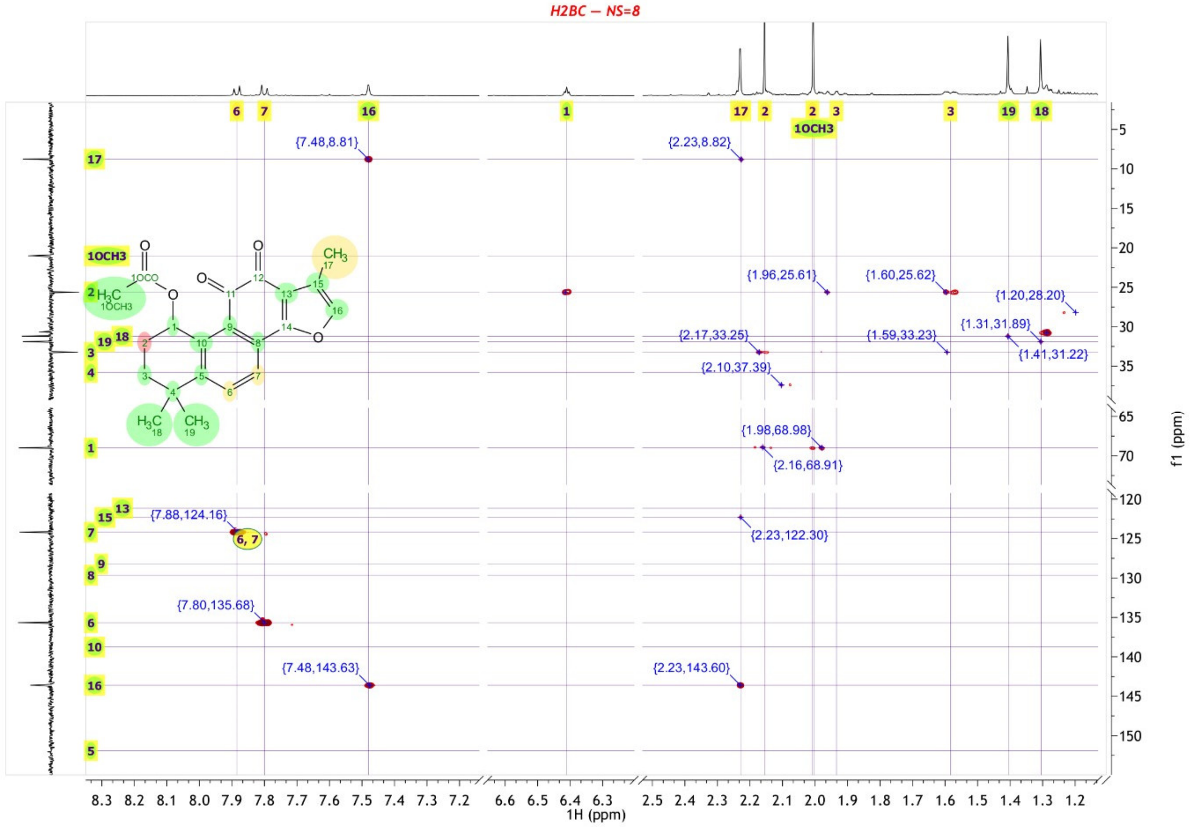

^{1}H-^{13}C HMBC 8 Hz NMR spectrum of compound 2 in CD_3OD, 25°C

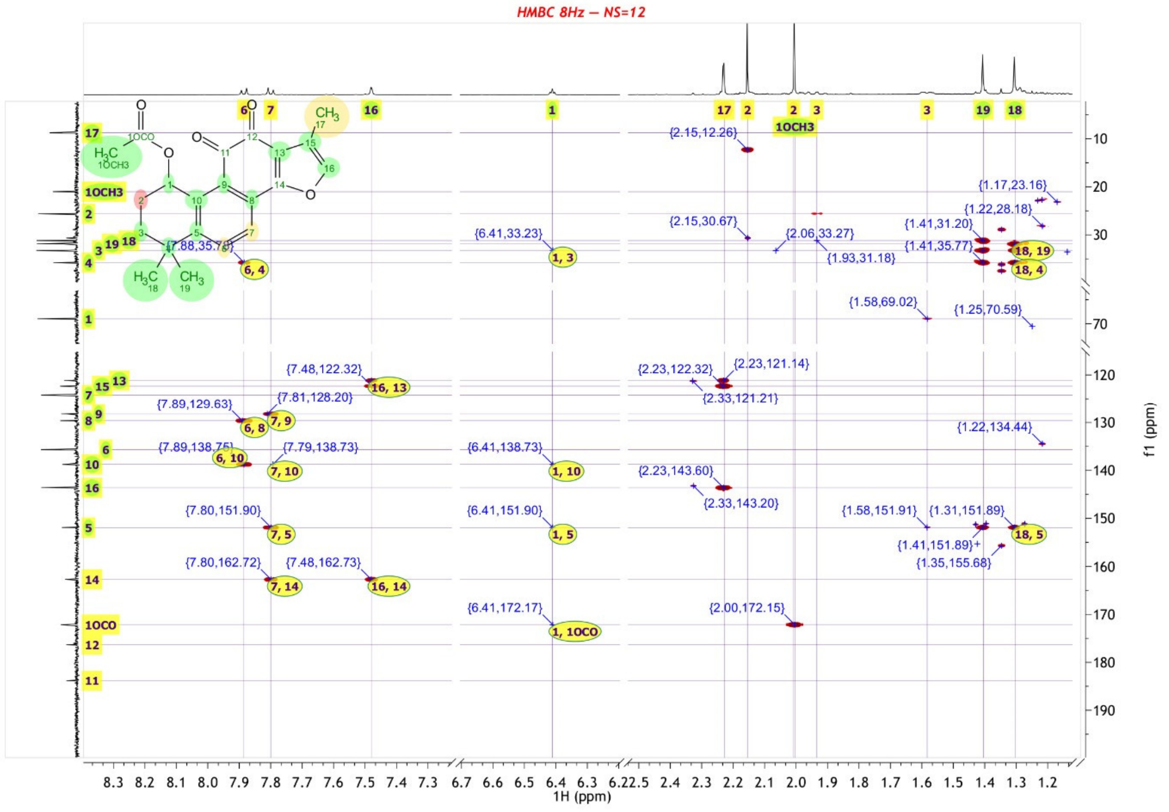

^{1}H selective-TOCSY spectrum at 6.41 ppm of compound 2 in CD_3OD, 25°C

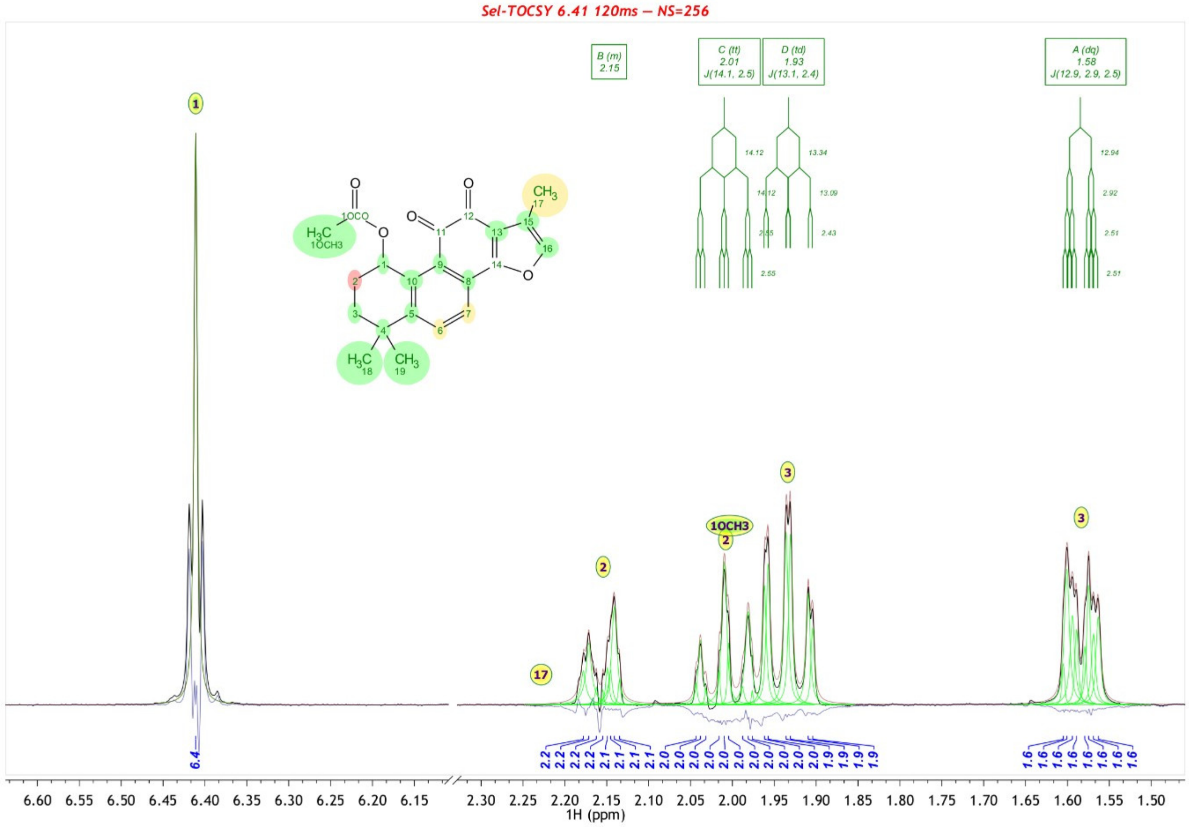

^{1}H (500 MHz) NMR spectrum of compound 3 in CD_3OD, 25°C

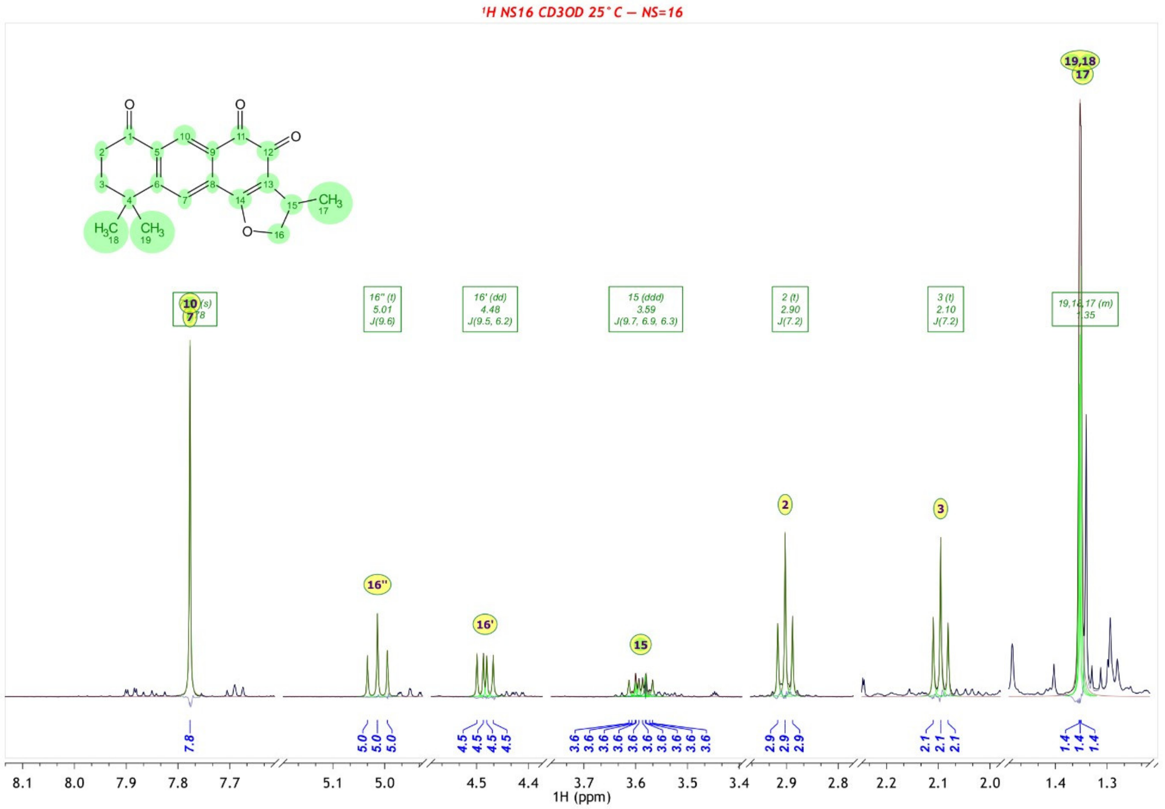

^{13}C (125 MHz) NMR spectrum of compound 3 in CD_3OD, 25°C

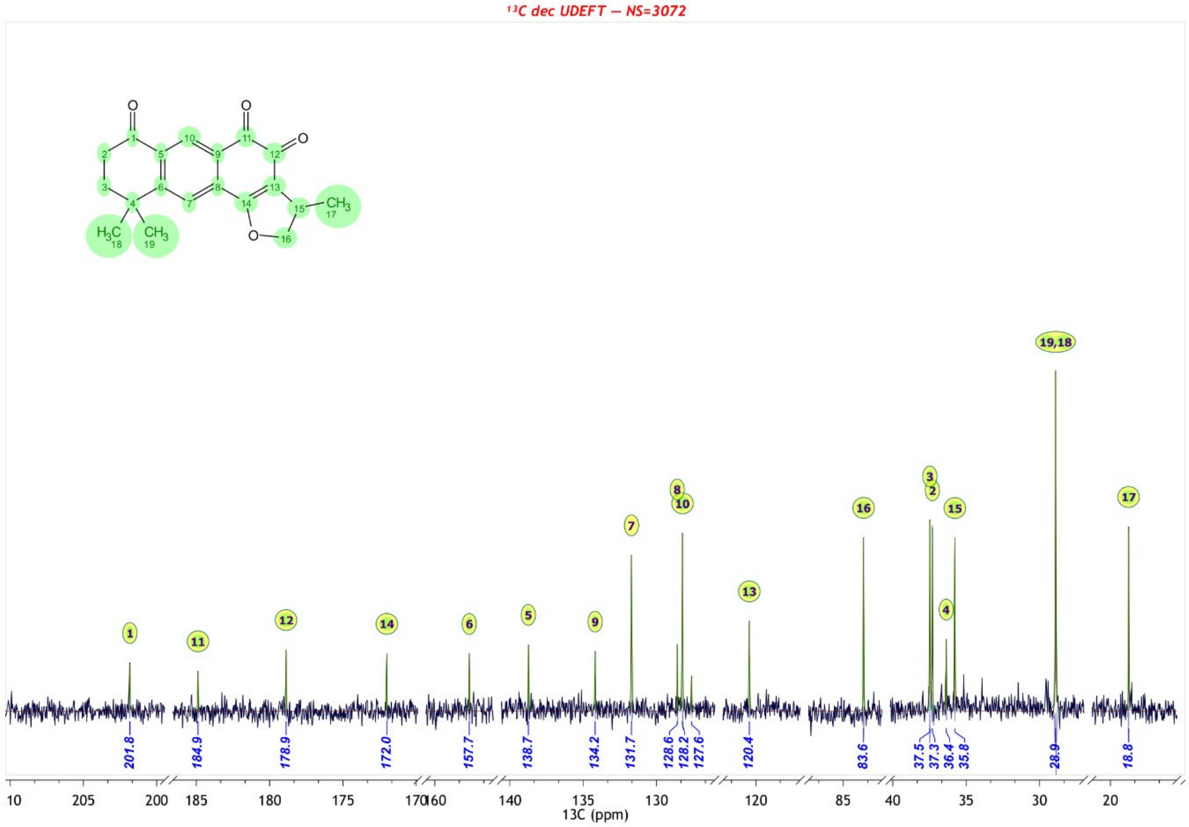

^1H-^1H COSY NMR spectrum of compound 3 in CD_3OD, 25°C

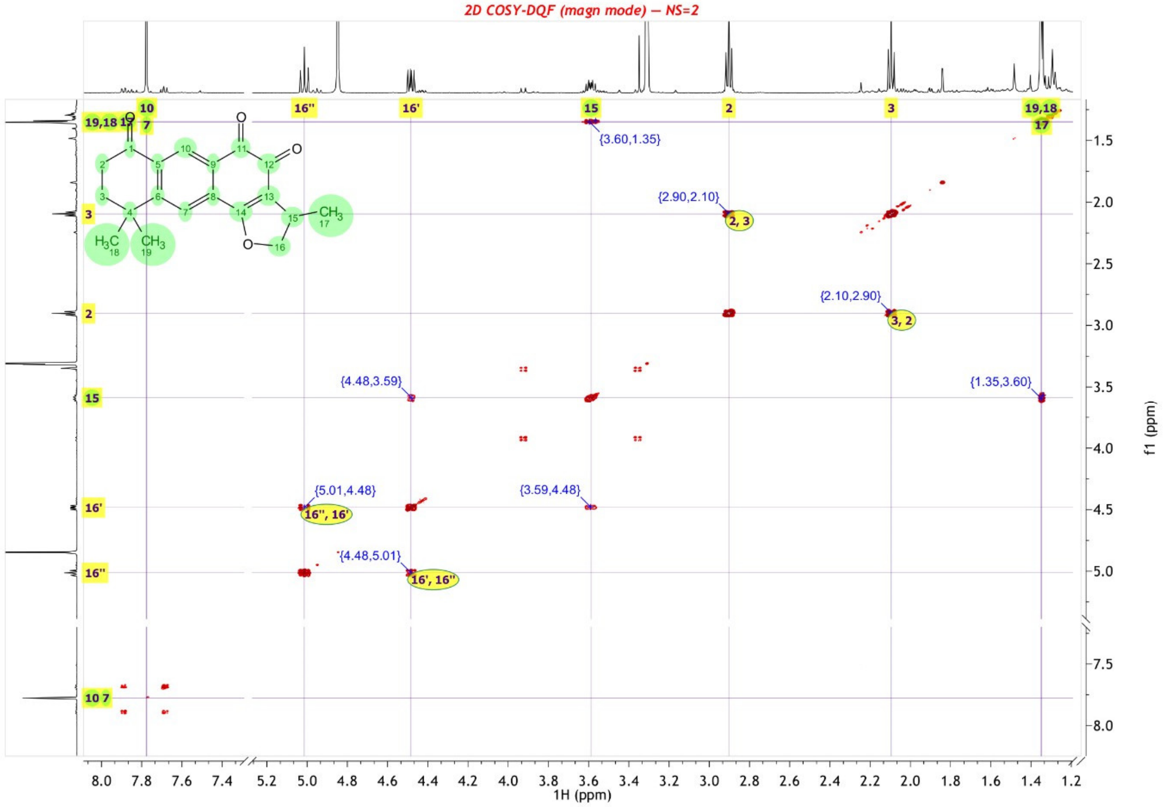

^1H-^1H TROESY NMR spectrum of compound 3 in CD_3OD, 25°C

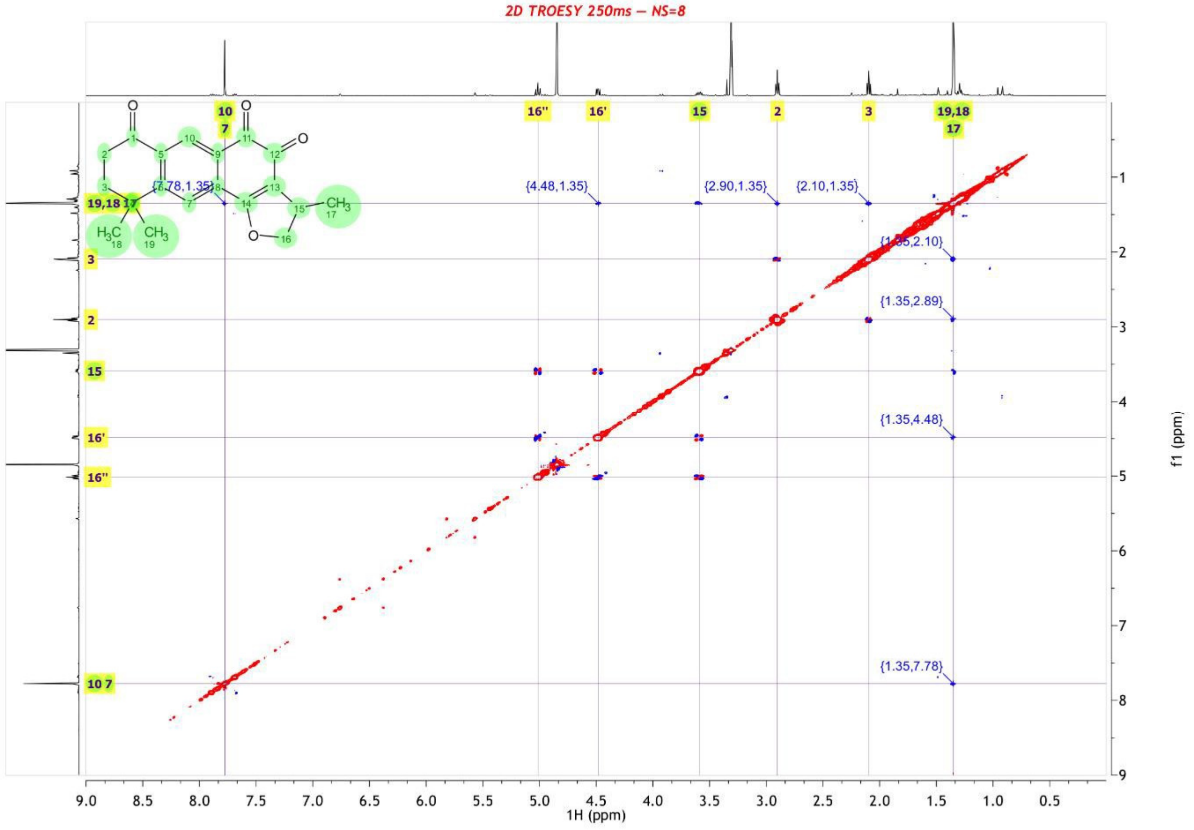

^{1}H-^{13}C HSQC NMR spectrum of compound 3 in CD_3OD, 25°C

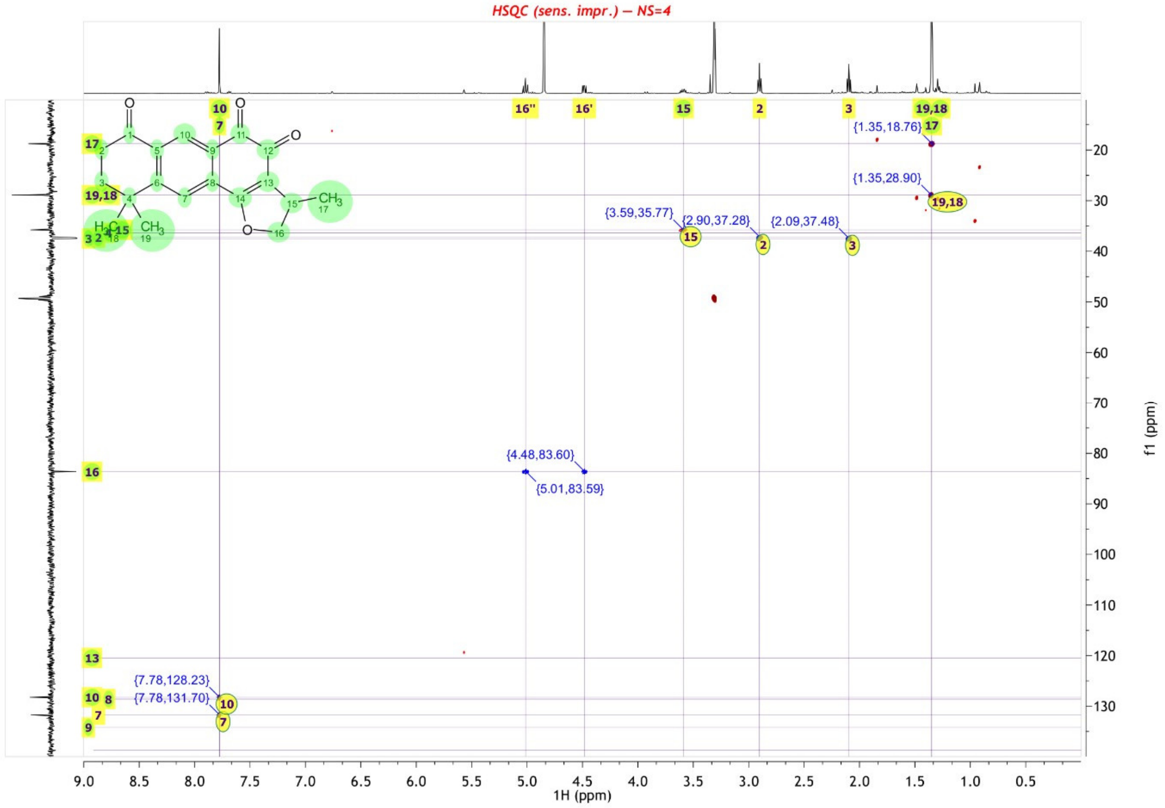

^{1}H-^{13}C H2BC NMR spectrum of compound 3 in CD_3OD, 25°C

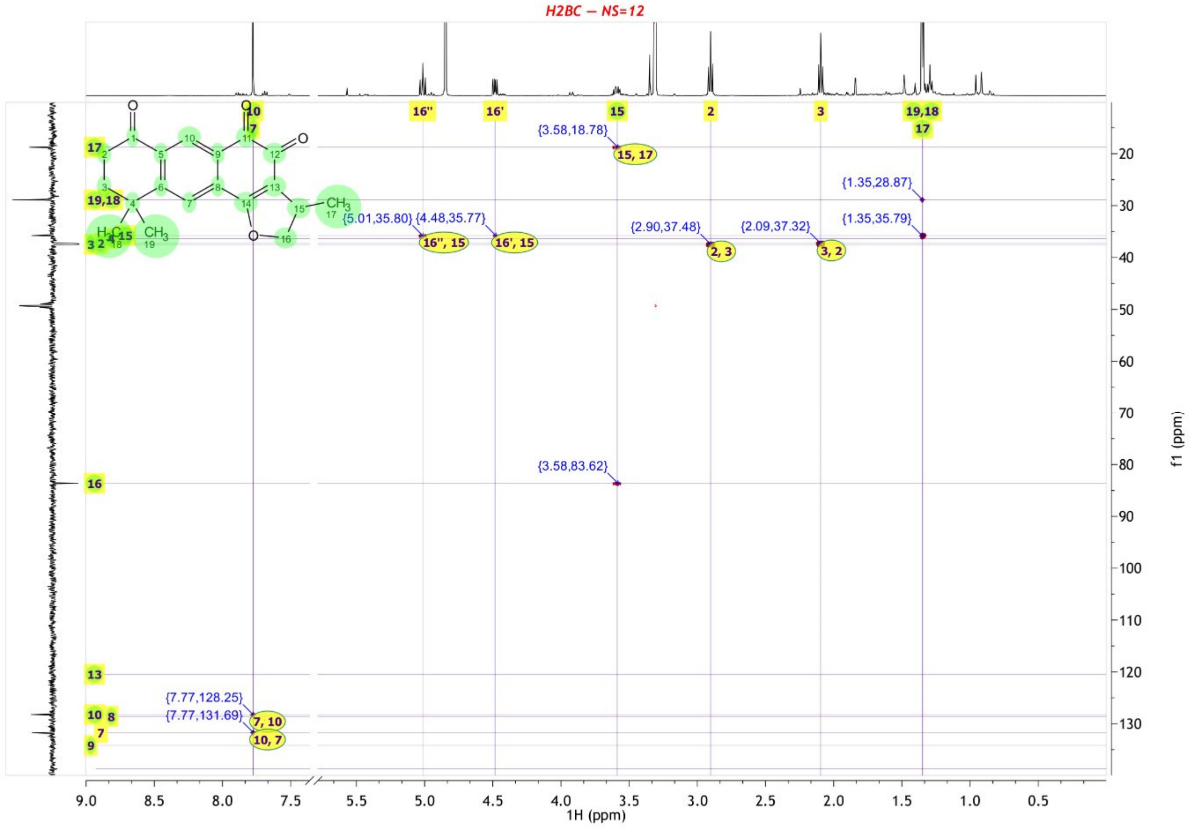

^{1}H-^{13}C HMBC 8 Hz NMR spectrum of compound 3 in CD_3OD, 25°C

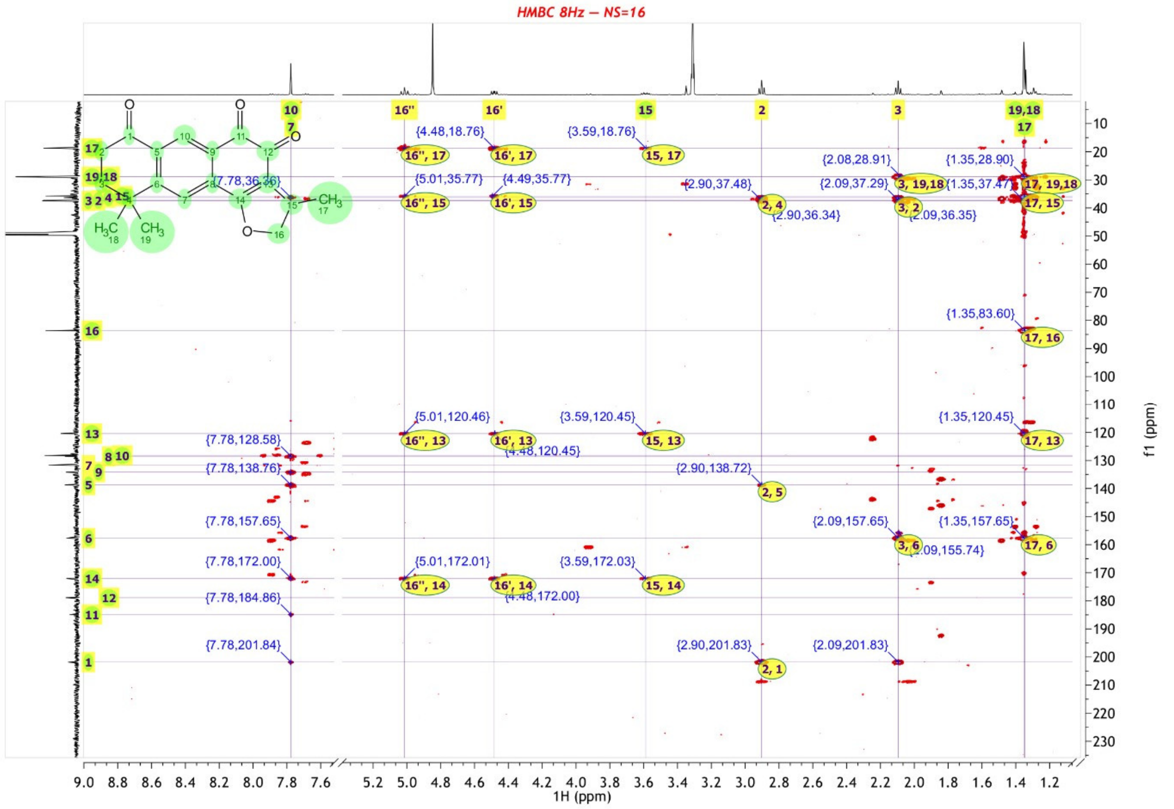

^{1}H-^{13}C HMBC 3 Hz NMR spectrum of compound 3 in CD_3OD, 25°C

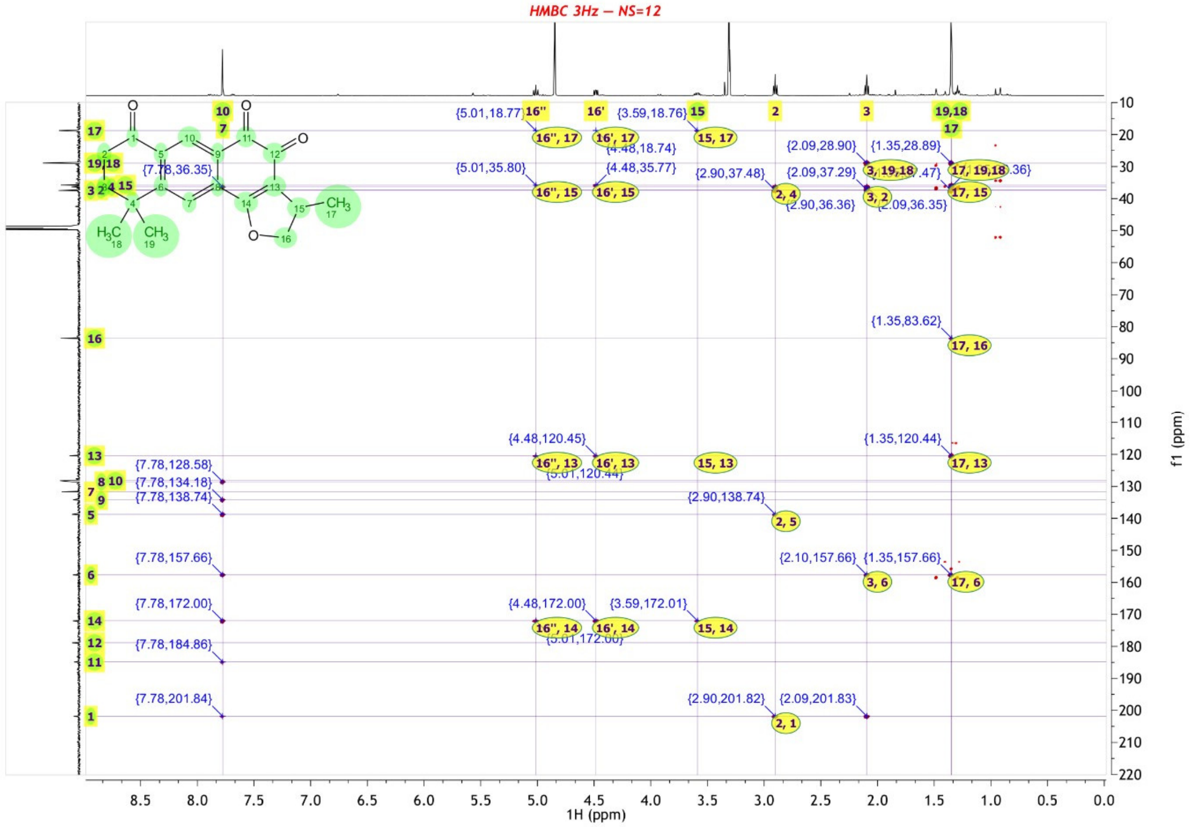

^{1}H selective-TOCSY spectrum at 5.01 ppm of compound 3 in CD_3OD, 25°C

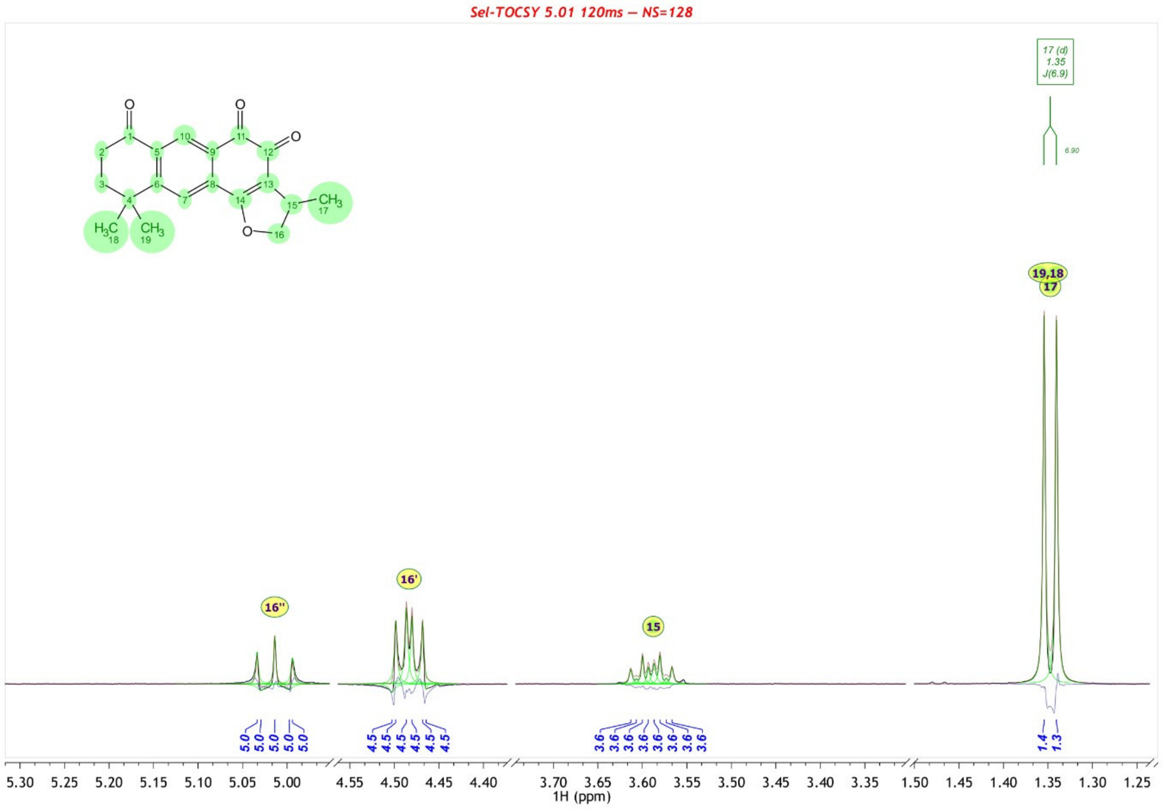

Supplement: Supplementary file 1 [file ijms-21-04475-s001.pdf]
